# Powerful t-tests in the presence of nonclassical measurement error

**DOI:** 10.1080/07474938.2024.2334166

**Published:** 2024-04-24

**Authors:** Dongwoo Kim, Daniel Wilhelm

**Affiliations:** a Department of Economics, Simon Fraser University, Burnaby, British Columbia, Canada; b Departments of Statistics and Economics, Ludwig-Maximilians-Universität München, Munich, Germany

**Keywords:** nonclassical measurement error, repeated measurement, linear regression, powerful t-tests, bootstrap

## Abstract

This article proposes a powerful alternative to the t-test of the null hypothesis that a coefficient in a linear regression is equal to zero when a regressor is mismeasured. We assume there are two contaminated measurements of the regressor of interest. We allow the two measurement errors to be nonclassical in the sense that they may both be correlated with the true regressor, they may be correlated with each other, and we do not require any location normalizations on the measurement errors. We propose a new maximal t-statistic that is formed from the regression of the outcome onto a maximally weighted linear combination of the two measurements. The critical values of the test are easily computed via a multiplier bootstrap. In simulations, we show that this new test can be significantly more powerful than t-statistics based on OLS or IV estimates. Finally, we apply the proposed test to a study of returns to education based on twin data from the UK. With our maximal t-test, we can discover statistically significant returns to education when standard t-tests do not.

## 1. Introduction

The presence of measurement error in regressors causes the variance of OLS and IV estimators to be larger than those in the case without measurement error. As a result, t-tests on the regression coefficients may suffer from poor power. This problem can be particularly severe when the measurement errors are nonclassical, i.e., when they depend on the true level of the regressor and/or when they depend on measurement errors of other measurements. In most economic applications in which the presence of measurement error is a first-order concern, neither of these two types of dependence can be ruled out.

For example, earnings have been shown to contain measurement error so that high-earners under-reported and low-earners over-reported true earnings (Bound et al., 2001). Therefore, measurement error must be nonclassical. In twins data such as that in Ashenfelter and Krueger (1994) and Bonjour et al. (2003), the twins’ reports on education have been argued to be mismeasured and nonclassical (e.g., Black et al., 2000; Hu and Sasaki, 2017). In this example, one would not want to rule out the possibility that the measurement errors of the twins are correlated because the twins are likely to possess correlated abilities to report correctly. In the literature on estimation of the skill production function (e.g., Attanasio et al., 2020a, 2020b; Cunha et al., 2010; Heckman et al. 2013), the measurements of skill production inputs are often elicited through the same data collection process; e.g., measurements of parental inputs may come from survey responses by the mother, which are responses recorded in short succession, on the same day, by the same interviewer. Therefore, mistakes in such responses are likely to be correlated. More generally, any study that uses repeated measurements of a latent variable is likely to incur correlated measurement errors. Black et al. (2000) provide additional economic examples for which there is evidence for the presence of nonclassical measurement errors.

In this article, we propose to combine two measurements of the unobserved, true regressor so as to construct a new (“maximal”) t-statistic that we show, in simulations, leads to a more powerful test compared to standard t-tests. Specifically, consider the linear regression

$$\begin{array}{l} Y=\beta X^{*}+\varepsilon , \end{array}$$

where *Y* is an outcome variable and X^*^ an unobserved regressor. Suppose we observe two measurements *X* and *Z* of X^*^ that are uncorrelated with the regression error *ε*. We want to test the null hypothesis of no effect,

$$\begin{array}{l} H_{0}:\beta =0\;\text{vs.}\;H_{1}:\beta \neq 0 \end{array}$$

using data on *Y*, *X*, and *Z*. If we knew a priori that the measurement errors in *X* and *Z* were classical and that the one in *Z* had a larger variance than that in *X*, then a t-test based on the OLS regression of *Y* on *X* would lead to a more powerful test than one based on the OLS regression of *Y* on *Z*. In that case, one might, for example, discard the noisier measurement *Z* and report only results from the regression of *Y* on *X*. The problem with this approach is that it crucially depends on the assumption of classical measurement error and on the knowledge of which measurement is less contaminated. Both of these are typically difficult, if not impossible, to justify.

The new “maximal” t-statistic proposed in this article combines the two measurements in a data-driven fashion to make the t-statistic as large as possible. In the example above with classical and independent measurement errors, the maximal t-statistic will automatically put more weight on the precise measurement *X* relative to the noisy measurement *Z* without the user having to know a priori which one is more precise. However, for the new test to be valid, we do not need to assume the measurement errors to be classical, i.e., they may depend on the true level of the regressor and the errors in the two measurements may depend on each other. We will see in the simulations that power gains relative to standard t-tests may be particularly large in the presence of nonclassical measurement error.

To develop the new maximal t-test, we first introduce the class of IV estimands from a regression of *Y* onto an arbitrary linear combination of *X* and *Z*, using a possibly different linear combination as the instrument. Standard OLS and IV estimands are special cases of this class, corresponding to particular weights in the linear combinations. The t-statistic derived from the estimator of the general estimand can then be optimized over the four weights (two in each of the two linear combinations of *X* and *Z*), leading to our maximal t-statistic. By maximizing over the weights, we test the null of no effect for all weights simultaneously, whereas standard OLS and IV t-tests test the null only for specific choices of those weights.

We show that, with homoskedastic regression errors, there exists a closed-form solution for the maximal weights in the linear combinations, leading also to a closed-form expression of the maximal t-statistic. With more general heteroskedasticity-robust variance estimators, such closed-form expressions may not exist. It is then immediate to see that the maximal t-statistic is, in general, strictly larger than standard OLS- or IV-based t-statistics. One might therefore wonder whether it is possible to construct a more powerful test based on this test statistic. We propose a multiplier bootstrap procedure for the calculation of critical values. Extensive Monte Carlo experiments confirm that the resulting maximal t-test is indeed very powerful. In all our simulation scenarios, the maximal t-test dominates all other OLS- and IV-based tests, sometimes by substantial margins, or dominates all but one, where this latter one performs similarly well.

We also show that it is not possible to improve Black et al. (2000)’s OLS and IV-based identification bounds on *β* by considering linear combinations of *X* and *Z*. The maximal t-test approach does therefore not lead to improved inference procedures in the more general hypothesis testing problem of

$$\begin{array}{l} H_{0}:\beta =b\;\text{vs.}\;H_{1}:\beta \neq b \end{array}$$

for some value *b*∈*ℝ*.

We illustrate the empirical usefulness of our new maximal t-test by revisiting a study of returns to schooling using twins data from the UK (Bonjour et al., 2003). Amin (2011) removed outliers from the same data set and found that the estimated returns in the original paper may be too large and that they may not be statistically significant. By employing our maximal t-test, which remains powerful even in the presence of nonclassical ME and in small samples, we can recover statistically significant returns to education when standard t-tests, as in Amin (2011) do not (e.g., even after removing outliers). We, therefore, view our results as providing robustness in support of the original results in Bonjour et al. (2003).

There is a large literature on identification and estimation of parametric and nonparametric models with measurement error. See, for example, the surveys by Aigner et al. (1984), Bound et al. (2001), Chen et al. (2011), and Schennach (2013). Valid inference in parametric models is usually rather standard once an identification argument and a consistent estimator have been provided. For example, the general approach by Schennach (2014) allows GMM-like moment conditions to depend on observables and unobservables and shows how to convert them into equivalent moment conditions that depend only on observables. The testing of general hypotheses then proceeds by using standard GMM inference procedures. However, we are not aware of any work trying to *improve* measurement error-robust inference methods by exploiting information in multiple measurements, which is the subject of this article.

## 2. Maximal combination of measurements

In this section, we explore the possibility of maximizing the t-statistic by optimally combining two measurements of the latent regressor. First, we consider testing the hypothesis of no effect and then the more general hypothesis for pre-specified effect size. We show that, in the former problem, the t-statistic based on maximally weighted linear combinations is, in general, larger than those based on the OLS and IV counterparts, but also that this is not possible in the latter problem.

### 2.1. Testing the hypothesis of no effect

To explain the main idea of this section, consider first the following simple regression model for a scalar outcome *Y* and a scalar regressor X^*^ satisfying

(1) $$\begin{array}{l} Y=\beta X^{*}+\varepsilon . \end{array}$$

The outcome *Y* is observed, but X^*^ is not. Instead, we observe two measurements *X* and *Z* of X^*^ that may contain measurement errors (MEs) $U:=X-X^{*}$ and $V:=Z-X^{*}$. Defining ME to be additive is without loss of generality because we will neither impose any localization restriction on the ME nor assume that it is independent of the true regressor X^*^. We will not even restrict the dependence among the two MEs *U* and *V*. We assume that E(εX)=E(εZ)=0, which is implied, for instance, if the latent regressor is exogenous ($E(\varepsilon X^{*})=0$) and the measurement errors are nondifferential (E(εU)=E(εV)=0).

Suppose we are interested in testing

(2) $$\begin{array}{l} H_{0}:\beta =0\;\text{vs.}\;H_{1}:\beta \neq 0. \end{array}$$

The OLS estimand of a regression of *Y* on *X* and the IV estimand from a regression of *Y* on *X* using *Z* as instrument are

$$\begin{array}{ll} \beta_{OLS} & :=\frac{E[YX]}{E[X^{2}]}=\beta \frac{E[(X^{*}{)}^{2}]+E[X^{*}U]}{E[(X^{*}{)}^{2}]+2E[X^{*}U]+E[U^{2}]}\\ \beta_{IV} & :=\frac{E[YZ]}{E[XZ]}=\beta \frac{E[(X^{*}{)}^{2}]+E[X^{*}V]}{E[(X^{*}{)}^{2}]+E[X^{*}U]+E[X^{*}V]+E[UV]} \end{array}$$

Under the null, $\beta_{OLS}=\beta_{IV}=0$, so the standard t-tests based on the OLS or IV estimators are asymptotically at correct level regardless of the dependence structure of the measurement system (i.e., the values of $E[X^{*}U],E[X^{*}V],$ and *E*[*UV*]).^1^ However, the power of these two tests may be poor for two reasons: under any fixed and arbitrarily large alternative *β*≠0, at least one of the estimands *β*_*OLS*_ and *β*_*IV*_ is close to zero when (a) *E*[X^*^U] or *E*[X^*^V] is negative and large in absolute values or (b) one of *E*[*U*^2^], *E*[X^*^U], *E*[X^*^V], *E*[*UV*] is large. Since the OLS and IV estimands depend on those quantities in different ways, the resulting t-tests may possess poor power under different data-generating processes. Similarly, one could construct t-tests based on OLS and IV estimators with the roles of *X* and *Z* switched. Which one of these four tests is most powerful depends on how strongly correlated *X* and *Z* are with X^*^ and the dependence structure in the measurement system. Since these quantities are neither known nor estimable without further assumptions, it is not possible to know a priori which of these tests should be used. The purpose of this article is to propose a new test that is powerful regardless of the configuration of these unknown quantities.

To start the discussion of what would be powerful tests in this setup, first consider the hypothetical case in which X^*^ is observed. In that case, the t-test based on OLS of *Y* on X^*^ is optimal: it is uniformly most powerful and unbiased in finite samples if regression errors are normal with known variance and the regressors are fixed (Section 7.6, Lehmann and Romano 2005). In large samples, the t-test is asymptotically optimal under regularity conditions without normality (Section 13.3, Lehmann and Romano 2005). In our setup, this test can of course not be implemented as a test can only depend on the observables *Y*, *X*, and *Z*.

Next, consider tests that only use second moments of the observables, specifically *E*[(*X*, *Z*)^′^*Y*]. For the purpose of this paragraph, suppose the observables have been demeaned. Under the null hypothesis, $E[(X,Z{)}^{\prime }Y]=0$, while under the alternative $E[(X,Z{)}^{\prime }Y]=E[(X,Z{)}^{\prime }X^{*}]\beta .$ We further assume that the sample average *E*_*n*_[(*X*, *Z*)^′^*Y*] is normally distributed with mean $E[(X,Z{)}^{\prime }X^{*}]\beta$ and known variance Σ. Then, the likelihood ratio test of *H*_0_ versus *H*_1_ is the uniformly most powerful unbiased test. Given the normality of *E*_*n*_[(*X*, *Z*)^′^*Y*], the likelihood ratio is an increasing function of

$$\begin{array}{l} E_{n}{\left[\begin{array}{c} XY\\ ZY \end{array}\right]}^{\prime }\Sigma^{-1}E_{n}\left[\begin{array}{c} XY\\ ZY \end{array}\right]-{\left(E_{n}\left[\begin{array}{c} XY\\ ZY \end{array}\right]-E\left[\begin{array}{c} XX^{*}\\ ZX^{*} \end{array}\right]\beta \right)}^{\prime }\Sigma^{-1}\left(E_{n}\left[\begin{array}{c} XY\\ ZY \end{array}\right]-E\left[\begin{array}{c} XX^{*}\\ ZX^{*} \end{array}\right]\beta \right). \end{array}$$

Thus, the likelihood ratio test rejects for large absolute values of

$$\begin{array}{l} E{\left[\begin{array}{c} XX^{*}\\ ZX^{*} \end{array}\right]}^{\prime }\Sigma^{-1}E_{n}\left[\begin{array}{c} XY\\ ZY \end{array}\right]. \end{array}$$

This expression can be viewed as weighting two OLS and/or IV estimators (for instance, *E*_*n*_[*XY*]∕*E*_*n*_(*X*^2^) and *E*_*n*_[*ZY*]∕*E*_*n*_(*Z*^2^)), where the weights depend on *E*[XX^*^] and *E*[ZX^*^]. Since these two quantities are neither known nor estimable without further assumptions, the likelihood ratio test is not feasible (we will refer to it as the “oracle” test). It does, however, suggest that t-tests based on optimally weighted OLS or IV estimators may be more powerful than the standard t-tests based on a single OLS or IV estimator.

Therefore, we propose a new “maximal t-test” that is based on weighted combinations of OLS and IV estimators. To introduce this test, for any a,b∈R, let W(a,b):=aX+bZ be an arbitrarily weighted linear combination of the two measurements *X* and *Z*. For $\omega :=(\omega_{1},\omega_{2},\omega_{3},\omega_{4}{)}^{\prime }\in R^{4}$, consider the IV estimand from the regression of *Y* onto the linear combination *W*(*ω*_3_, *ω*_4_) using a possibly different linear combination *W*(*ω*_1_, *ω*_2_) as instrument,

(3) $$\begin{array}{l} \beta (\omega ):=\frac{E[W(\omega_{1},\omega_{2})Y]}{E[W(\omega_{1},\omega_{2})W(\omega_{3},\omega_{4})]}. \end{array}$$

The OLS estimand of regressing *Y* onto *X* (*Z*) corresponds to β(1,0,1,0) (β(0,1,0,1)) and the IV estimand of regressing *Y* onto *X* (*Z*) using *Z* (*X*) as IV corresponds to β(1,0,0,1) (β(0,1,1,0)).

The null implies that *β*(*ω*) = 0 for all *ω*∈*ℝ*^4^ and, as long as $E[(X^{*}{)}^{2}]+E[X^{*}V]\neq 0$ and $E[(X^{*}{)}^{2}]+E[X^{*}U]\neq 0$, one can easily show that (4) is equivalent to

(4) $$\begin{array}{l} H_{0}:\underset{\omega \in R}{\sup}|\beta (\omega )|=0\;\;\text{vs.}\;\;H_{1}:\;\underset{\omega \in R}{\sup}|\beta (\omega )|\neq 0. \end{array}$$

If the regression errors are homoskedastic, we standardize the estimand by

$$\begin{array}{l} \sigma^{2}(\omega ):=\frac{\sigma_{\varepsilon }^{2}(\omega )E\left[W(\omega_{1},\omega_{2}{)}^{2}\right]}{{\left(E[W(\omega_{1},\omega_{2})W(\omega_{3},\omega_{4})]\right)}^{2}} \end{array}$$

where $\sigma_{\varepsilon }^{2}(\omega ):=E[\varepsilon (\omega {)}^{2}]$ with $\varepsilon (\omega ):=Y-\beta (\omega )W(\omega_{3},\omega_{4})$, is the residual variance. Given the reformulation of the null in (4), we consider the maximal t-ratio $\underset{\omega \in R}{\sup}|t(\omega )|$ with

(5) $$\begin{array}{l} t(\omega ):=\frac{\beta (\omega )}{\sigma (\omega )} \end{array}$$

because it imposes all restrictions under the null, unlike the OLS and IV estimands which only impose the null for one specific choice of *ω*.

Assumption 1.

- E[εX]=E[εZ]=0 *,* $E[X^{*}X]\neq 0$ *and* $E[ZX^{*}]\neq 0$
- $Var(X^{*})>0$ *,* *Var*(*X*) > 0 *,* *Var*(*Z*) > 0 *,* *Var*(*ε*) > 0
- |Corr(X,Z)|≠1 *and* |Corr(Y,X−Z)|≠1

Notice that part (i) and (ii) of this assumption allows for endogeneity of the latent regressor ($E[\varepsilon X^{*}]\neq 0$), requiring only that the measurements *X* and *Z* are exogenous. More importantly, the assumption does not restrict the covariances $\left(E[X^{*}U],E[X^{*}V],E[UV]\right)$. Part (i) also requires the measurements *X* and *Z* to be correlated with X^*^. So, when viewing (Y,X,Z) as three measurements of X^*^, our analysis requires that two of them are correlated with X^*^ to be able to test whether the third one is also correlated with X^*^. Part (iii) requires that the two measurements are not linearly dependent and that *Y* is not perfectly correlated with the difference between the two MEs.

Theorem 1. *Suppose model (1) and Assumption 1 hold. Then, $\omega^{*}:=\arg\underset{\omega \in R^{4}}{\sup}|t(\omega )|$ is of the form $\omega^{*}=(a^{*},1-a^{*},a^{*},1-a^{*})$ with*

$$\begin{array}{l} a^{*}:=\frac{E[XZ]E[ZY]-E[Z^{2}]E[XY]}{E[X(Z-X)]E[ZY]+E[Z(X-Z)]E[XY]} \end{array}$$

*with the possibility of $|a^{*}|=\infty$ if the denominator is equal to zero.*

Since the maximal elements $\left(\omega_{3}^{*},\omega_{4}^{*}\right)$ are equal to $\left(\omega_{1}^{*},\omega_{2}^{*}\right)$, the estimand for the maximal weights becomes that of the OLS regression of *Y* onto $W(a^{*},1-a^{*})$:

$$\begin{array}{l} \beta (\omega^{*})=\frac{E[W(a^{*},1-a^{*})Y]}{E[W(a^{*},1-a^{*}{)}^{2}]}. \end{array}$$

Notice that, in general, the weight a^*^ may be outside [0, 1]. In fact, it may be equal to ±∞. Interestingly, the weight depends only on observable quantities based on the distribution of (Y,X,Z), but leads to the largest possible t-ratio regardless of the unknown measurement covariance structure $\left(E[X^{*}U],E[X^{*}V],E[UV]\right)$ of the measurement system. In this sense, the maximal t-ratio adapts to the unknown measurement covariance structure without the researcher having to place a priori restrictions on those covariances.

In the special case in which *X* contains no information about the latent regressor X^*^ (i.e., *X* is uncorrelated with X^*^), *X* is uncorrelated with the ME in *Z*, and *E*[*Xε*] = 0, then E[XZ]=E[XY]=0 and the maximal weight becomes $a^{*}=0$. This weight corresponds to OLS regression of *Y* onto *Z*, ignoring the second measurement *X*. On the other hand, when *Z* contains no information about the latent regressor X^*^, *Z* is uncorrelated with the ME in *X*, and *E*[*Zε*] = 0, then E[XZ]=E[ZY]=0 and the maximal weight becomes $a^{*}=1$. This weight corresponds to the OLS regression of *Y* onto *X*, ignoring the second measurement *Z*. Similarly, using the inverse function theorem one can show that there exists an *a* such that β(a,1−a,a,1−a) equals the IV estimand (for a regression of *Y* onto *X* (*Z*), using *Z* (*X*) as an instrument), but the expression for that *a*-value is quite complicated.

Since the maximal weight ω^*^ can attain any value on the extended real line, it is immediately clear that the maximal t-ratio $\left|t(\omega^{*})\right|$ is not smaller than the absolute value of the corresponding OLS and the IV t-ratios. Therefore, there exist data-generating processes for which the maximal t-ratio is strictly larger than any of them and one might expect that imposing all restrictions of the null by testing whether the maximal t-ratio is equal to zero might translate into favorable power properties of the test. Section 4 confirms this intuition.

Since ω^*^ possesses a closed-form solution, the maximal t-ratio also does. For example, when $|a^{*}|<\infty$, then the maximal t-ratio can be written as

$$\begin{array}{l} t(\omega^{*})=\text{sign}(C)\sqrt{\frac{A}{B}} \end{array}$$

with

$$\begin{array}{ll} A & :=E\left[{\left(ZE[XY]-XE[ZY]\right)}^{2}\right]\\ B & :=A-E[Y^{2}]\left((E[XZ]{)}^{2}-E[Z^{2}]E[X^{2}]\right)\\ C & :=E[X(Z-X)]E[ZY]+E[Z(X-Z)]E[XY] \end{array}$$

Under the null, the t-statistic is independent of the weight, so whether the weight is finite or infinite does not change its functional form. Under alternatives *β*≠0, the case $|a^{*}|=\infty$ is unlikely and occurs only in extreme cases in which the MEs are perfectly correlated with the true regressor X^*^ or the regression error *ε* (see Lemma 1 in Appendix A).

Now suppose there are additional, correctly measured, exogenous regressors *R*, *E*(*εR*) = 0, so that

(6) $$\begin{array}{l} Y=\beta X^{*}+\gamma^{\prime }R+\varepsilon . \end{array}$$

Denote by $\tilde{X}$, $\tilde{Z}$, $\tilde{Y}$ the residuals of regressions of *X*, *Z*, *Y* onto *R* and let $\tilde{W}(a,b):=a\tilde{X}+b\tilde{Z}$. Define $\tilde{\beta }(\omega )$ like *β*(*ω*), replacing *W*(*a*, *b*) by $\tilde{W}(a,b)$, and ${\tilde{\sigma }}^{2}(\omega )$ like *σ*^2^(*ω*), replacing Y,W(a,b),β(ω) by $\tilde{Y},\tilde{W}(a,b),\tilde{\beta }(\omega )$. The t-ratio is then defined as

(7) $$\begin{array}{l} \tilde{t}(\omega ):=\frac{\tilde{\beta }(\omega )}{\tilde{\sigma }(\omega )}. \end{array}$$

The OLS estimand of regressing *Y* onto (*X*, *R*) corresponds to $\tilde{\beta }(1,0,1,0)$, the IV estimand regressing *Y* onto (*X*, *R*) using (*Z*, *R*) as IVs corresponds to $\tilde{\beta }(1,0,0,1)$.

The maximal t-ratio and its maximizer can then be found using the result in Theorem 1:

Corollary 1. *Suppose (6) and Assumption 1 hold with (Y,X,Z) replaced by $\left(\tilde{Y},\tilde{X},\tilde{Z}\right)$. Then, ${\tilde{\omega }}^{*}:=\underset{\omega \in R^{4}}{\sup}|\tilde{t}(\omega )|$ is of the form ${\tilde{\omega }}^{*}=({\tilde{a}}^{*},1-{\tilde{a}}^{*},{\tilde{a}}^{*},1-{\tilde{a}}^{*})$ with*

$$\begin{array}{l} {\tilde{a}}^{*}:=\frac{E[\tilde{X}\tilde{Z}]E[\tilde{Z}\tilde{Y}]-E[{\tilde{Z}}^{2}]E[\tilde{X}\tilde{Y}]}{E[\tilde{X}(\tilde{Z}-\tilde{X})]E[\tilde{Z}\tilde{Y}]+E[\tilde{Z}(\tilde{X}-\tilde{Z})]E[\tilde{X}\tilde{Y}]} \end{array}$$

*with the possibility of $|{\tilde{a}}^{*}|=\infty$ if the denominator is equal to zero.*

The t-ratios defined in (5) and (7) use asymptotic variance expressions for homoskedastic regression errors. We now consider conditional heteroskedasticity in the regression model (6), i.e., when the conditional variance of the regression error given X^*^ is not independent of X^*^. In this case, we are interested in the t-ratio

$$\begin{array}{l} \bar{t}(\omega ):=\frac{\tilde{\beta }(\omega )}{\bar{\sigma }(\omega )}, \end{array}$$

where the variance is

$$\begin{array}{l} {\bar{\sigma }}^{2}(\omega ):=\frac{E[\tilde{\varepsilon }(\omega {)}^{2}\tilde{W}(\omega_{1},\omega_{2}{)}^{2}]}{(E[\tilde{W}(\omega_{1},\omega_{2})\tilde{W}(\omega_{3},\omega_{4})]{)}^{2}} \end{array}$$

with $\tilde{\varepsilon }(\omega ):=\tilde{Y}-\tilde{\beta }(\omega )\tilde{W}(\omega_{3},\omega_{4})$.

Without any further restrictions on the form of heteroskedasticity, there does not in general exist a closed-form expression for the maximal weights or the maximal t-ratio. However, we can show that the four-dimensional optimization problem over *ω* can be reduced to a two-dimensional one, which simplifies implementation significantly.

Theorem 2. *Suppose model (6) and Assumption 1 hold with (Y,X,Z) replaced by $\left(\tilde{Y},\tilde{X},\tilde{Z}\right)$. Then, for any $\omega :=(\omega_{1},\omega_{2},\omega_{3},\omega_{4})\in R^{4}$, there exist $a_{1},a_{2}\in R$ such that, for $\omega^{\prime }:=(a_{1},1-a_{1},a_{2},1-a_{2})$,*

$$\begin{array}{l} \bar{t}(\omega )=\bar{t}(\omega^{\prime }). \end{array}$$

Remark 1. In principle, one could construct a test statistic for *H*_0_ using more complicated, nonlinear combinations of *X* and *Z* rather than the linear combinations as in the definition of *W*(*a*, *b*). For example, one could consider $W(a,b):=a^{\prime }p(X)+b^{\prime }q(Z)$, where $a,b\in R^{K}$, and *p*(⋅) and *q*(⋅) are *K*-dimensional vectors of basis functions (e.g., polynomials or splines). The results of this section can easily be extended to this case. However, allowing for interaction terms between the basis functions of *X* and *Z* would require more involved arguments, and we leave these for future work. In this article, we focus on linear combinations because we place more importance on the practical usefulness of the method and because we want to avoid the maximal t-statistic depending on higher-order moments of (Y,X,Z) which would make it more sensitive to outliers.

### 2.2. Testing general hypotheses

In this section, we consider testing the more general hypothesis

(8) $$\begin{array}{l} H_{0}:\;\beta =b\;\text{vs.}\;H_{1}:\;\beta \neq b \end{array}$$

for some *b*∈*ℝ* in the simple regression model (1). Under the null of no effect, (2), the estimand *β*(*a*) considered in the previous section is zero for all weights *a*. This is not the case under the more general null (8) because *β* is not identified under Assumption 1. However, one can construct bounds for *β* based on observable quantities by imposing further assumptions on correlations between MEs and X^*^. To describe these bounds, consider the following notation. Because of Theorem 2, it suffices to consider linear combinations of measurements with weights *a* and 1−*a*, so we simplify the notation to W(a):=aX+(1−a)Z. Let

$$\begin{array}{l} \beta_{OLS}(a):=\frac{E[W(a)Y]}{E[W(a{)}^{2}]} \end{array}$$

be the OLS estimand of a regression of *Y* on *W*(*a*), and

$$\begin{array}{l} \beta_{OLS-INV}(a):=\frac{E[Y^{2}]}{E[W(a)Y]} \end{array}$$

that of the reverse regression. Similarly, the IV estimand from a regression of *Y* on *W*(*a*) using *W*(*b*) as an instrument is

$$\begin{array}{l} \beta_{IV}(a,b):=\frac{E[W(b)Y]}{E[W(b)W(a)]}. \end{array}$$

Assumption 2. *
$E[\varepsilon X^{*}]=E[\varepsilon U]=E[\varepsilon V]=0$
*

Assumption 3. *$E[X^{*}X]>0$ and $E[X^{*}Z]>0$*

Assumption 4. *$E[X^{*}X]\geq E[XZ]>0$ and $E[X^{*}Z]\geq E[XZ]>0$*

Assumption 5. **E*[*UX*]≥0 and *E*[*VZ*]≥0*

Assumption 6. *$E[X^{*}U]\leq 0$ and $E[X^{*}V]\leq 0$*

Assumption 2 means X^*^ is exogenous and MEs are nondifferential. Assumptions 3 and 5 require that the MEs are not too severely correlated with X^*^ so that covariances between MEs and X^*^ do not dominate the variances of X^*^ and MEs, respectively. Assumption 4 is a stronger version of Assumption 3, which implies the correlation between X^*^ and either of mismeasured variables is stronger than the correlation between *X* and *Z*. Assumption 6 allows the MEs to be nonclassical and negatively correlated with $X^{*},$ which is likely to be the case when X^*^ has bounded support. Black et al. (2000) present detailed discussions on the above assumptions and show how to construct bounds on *β* under the assumptions.^2^ The assumptions are untestable and stronger than Assumption 1 but still substantially weaker than the classical ME assumption.

For the discussion in this section, suppose *β*≥0 (similar results hold for the case *β*≤0). First, the OLS estimand and the inverse OLS estimand form lower and upper bounds on the regression coefficient:

$$\begin{array}{l} \max_{a=0,1}\beta_{OLS}(a)\leq \beta \leq \min_{a=0,1}\beta_{OLS-INV}(a). \end{array}$$

The IV estimand provides an additional bound that may or may not be tighter than the OLS bound,

$$\begin{array}{l} \beta \leq \min_{a=0,1}\beta_{IV}(a,1-a). \end{array}$$

We now show that the bounds cannot be refined by considering linear combinations of measurements.

Theorem 3. *Suppose *β*≥0. Then:*

1. *Under Assumptions 2, 3, and 5,* *β*_*OLS*_(*a*)≤*β* *holds only for* a∈{0,1}.
2. *Under Assumptions 2, 3, and 6,* $\beta \leq \beta_{OLS-INV}(a)$ *holds only for* a∈[0,1].
3. *Under Assumptions 2 and 4,* $\beta \leq \beta_{IV}(a,1-a)$ *holds only for* a∈{0,1}.

The first and third statements of this theorem imply that it is impossible to refine the OLS and IV bounds using linear combinations of measurements. The proof shows that, for any a∉{0,1}, we can find a data-generating process satisfying the assumptions of the theorem for which the inequalities are violated. The second part of the theorem states that the inverse OLS bound holds for all a∈[0,1]. However, because the inverse OLS estimand is inversely related to a linear function of the weight *a*, it is easy to see that the tightest bound, $\min_{a\in [0,1]}\beta_{OLS-INV}(a)$, is achieved by either *a* = 0 or *a* = 1. Therefore, the inverse OLS bound also cannot be refined by considering linear combinations of the two measurements.

Under the null hypothesis of no effect (2), the estimand *β*(*a*) is equal to zero for all weights *a*, but it varies with *a* under the alternative. This is why it was possible to improve the power of the t-test by choosing *a* carefully. In the case of testing the general null (8), however, the estimand of interest is the set of possible *β* values consistent with the data,

$$\begin{array}{l} \Theta (a_{1},a_{2},a_{3}):=\left\{\beta \in R:\beta_{OLS}(a_{1})\leq \beta \leq \min\{\beta_{OLS-INV}(a_{2}),\beta_{IV}(a_{3},1-a_{3})\}\right\}. \end{array}$$

These sets contain the identified set for *β* and thus are valid outer sets for any $(a_{1},a_{2},a_{3})\in \{0,1{\}}^{3}$, but they are not for any other value of $\left(a_{1},a_{2},a_{3}\right)$. Therefore, we cannot take linear combinations of the measurements to tighten the identified set or improve inference, as in the approach of the previous section.

## 3. The maximal t-test

In this section, we propose a powerful t-test of the hypothesis (2), making use of the maximal combination of two measurements. We want to allow for heteroskedasticity-robust variance estimators in the construction of the t-statistic. As we have shown in Theorem 2, for the calculation of the maximal t-statistic, we do not have to consider general weights *ω* in *ℝ*^4^, but only weights of the form $\left(a_{1},1-a_{1},a_{2},1-a_{2}\right)$. A direct implementation of this result could consider the test statistic

$$\begin{array}{l} \max_{a=(a_{1},a_{2})\in R^{2}}\left|\hat{t}(a_{1},1-a_{1},a_{2},1-a_{2})\right|, \end{array}$$

where $\hat{t}$ is an estimator of *t* multiplied by $\sqrt{n}$. Under standard conditions, it is easy to show that $\hat{t}(\cdot )$ weakly converges to a Gaussian process, so that by the delta method, the maximal t-statistic converges to the supremum of that limiting process. Unfortunately, it is difficult to construct critical values from this process because its covariance function depends on the unknown data-generating process. We, therefore, propose a simple multiplier bootstrap method to calculate critical values.

Suppose we observe an i.i.d. sample $Y_{n}:=\{(Y_{i},X_{i},Z_{i}){\}}_{i=1}^{n}$ from the distribution of (Y,X,Z) and ignore additional, correctly measured covariates. We describe a procedure that applies to the numerical optimization of the t-ratio. Define a grid of weights $\omega_{1},\ldots ,\omega_{p}$ of the form $\omega_{j}=(a_{j,1},1-a_{j,1},a_{j,2},1-a_{j,2}{)}^{\prime }$ for some $(a_{j,1},a_{j,2}{)}^{\prime }\in R^{2}$. Let $W_{1ij}:=\omega_{j,1}X_{i}+\omega_{j,2}Z_{i}$, $W_{2ij}:=\omega_{j,3}X_{i}+\omega_{j,4}Z_{i}$, $\sigma_{j}^{2}:=E[\varepsilon_{i}^{2}W_{1ij}^{2}]$, and ${\hat{\sigma }}_{j}^{2}$ some estimator of $\sigma_{j}^{2}$. The notation *𝔼*_*n*_[⋅] denotes the average over the index 1≤i≤n, so for example $E_{n}[x_{ij}]=n^{-1}\sum_{i=1}^{n}x_{ij}$.

Our maximal t-statistic is defined as $\left|\hat{t}(\omega )\right|$ optimized over the grid $\omega_{1},\ldots ,\omega_{p}$:

$$\begin{array}{l} T:=\max_{\omega \in \{\omega_{1},\ldots ,\omega_{p}\}}\left|\hat{t}(\omega )\right|=\max_{1\leq j\leq p}\left|\frac{\sqrt{n}E_{n}[W_{1ij}Y_{i}]}{{\hat{\sigma }}_{j}}\right|. \end{array}$$

We now describe the construction of critical values using a multiplier bootstrap. Let $\{e_{i}^{b}{\}}_{i=1}^{n}$, b=1,…,B, be an i.i.d. sequence of standard normally distributed random variables that are independent of *𝒴*_*n*_ and the residual ${\hat{\varepsilon }}_{ij}:=Y_{i}-{\hat{\beta }}_{j}W_{2ij}$ where ${\hat{\beta }}_{j}$ is the IV estimator from a regression of *Y*_*i*_ onto *W*_2*ij*_ using *W*_1*ij*_ as instrument. We define the bootstrap statistic

$$\begin{array}{l} T^{b}:=\max_{1\leq j\leq p}\left|\frac{\sqrt{n}E_{n}[e_{i}W_{1ij}{\hat{\varepsilon }}_{ij}]}{{\hat{\sigma }}_{j}}\right|,\;b=1,\ldots ,B, \end{array}$$

and the bootstrap critical value

$$\begin{array}{l} c_{\alpha }:=\text{conditional }(1-\alpha )-\text{quantile of }T^{b}\text{ given the data }Y_{n}. \end{array}$$

We reject *H*_0_ if and only if $T>c_{\alpha }.$

The validity of this bootstrap critical value can be shown using the high-dimensional central limit theorem in Corollary 3.1 of Chernozhukov et al. (2013). It implies that the test has a limiting rejection probability under the null equal to the nominal level *α* and is consistent against any fixed alternative violating the null. The result requires only mild conditions on the data-generating process and, in particular, allows the vector *W*_1*ij*_ to grow at a rate that is an exponential function of the sample size. This means that we can consider grids for weights $\omega_{1},\ldots ,\omega_{p}$ of very large size *p*.

Remark 2. In simulations and empirical applications, we also consider a version of the maximal t-test in which we impose homoskedasticity ($E[\varepsilon_{i}^{2}|W_{1ij}=w]$ does not vary with *w*) by using the variance estimator ${\hat{\sigma }}_{j}^{2}={\hat{\sigma }}_{\varepsilon ,j}^{2}E_{n}[W_{1ij}^{2}]$ where ${\hat{\sigma }}_{\varepsilon ,j}^{2}$ is the sample variance of ${\hat{\varepsilon }}_{ij}$.

While clearly desirable, a full analysis of optimal tests in the current setup with latent variables is left for future work. In this article, we simply take inspiration from the infeasible likelihood ratio (“oracle”) test, which optimally weights OLS and IV estimators, and propose a feasible alternative that maximizes the t-ratio over all possible weights. In the next section, we show in simulations that this new test may indeed be significantly more powerful than existing alternatives.

## 4. Simulations

This section studies the finite sample performance of the maximal t-test described in Section 3. We consider the simple regression model in (1) with the two measurements *X* and *Z* generated from $X=X^{*}+U$ and $Z=X^{*}+V$ with

$$\begin{array}{l} \left[\begin{array}{l} X^{*}\\ U\\ V \end{array}\right]∼N\left(\left[\begin{array}{l} 0\\ 0\\ 0 \end{array}\right],\left[\begin{array}{lcc} 1 & \sigma_{X^{*}U} & \sigma_{X^{*}V}\\ \sigma_{X^{*}U} & \sigma_{U}^{2} & \sigma_{UV}\\ \sigma_{X^{*}V} & \sigma_{UV} & \sigma_{V}^{2} \end{array}\right]\right) \end{array}$$

and ε∼N(0,1) is independent of $\left(X^{*},U,V\right)$. The null hypothesis holds with *β* = 0. To generate alternatives, we increase *β* on a grid up to 0. 8. We define seven different scenarios in which we vary the parameters $\sigma_{U}^{2}$, $\sigma_{V}^{2}$, and *σ*_*UV*_. Table 1 defines those scenarios. We vary the covariances $\sigma_{X^{*}U}$ and $\sigma_{X^{*}V}$ in each scenario. We generate 1, 000 Monte Carlo samples of size *n* = 200.

**Table 1.** Parameter values in each scenario.

Scenario	$\sigma_{U}^{2}$, $\sigma_{V}^{2}$	*σ*_*U* *V*_	$\sigma_{X^{*}U}$	$\sigma_{X^{*}V}$
0	0,1	0	0	−0.3 or −0.5
1	2	0	−0.3 or −0.7	−0.3 or −0.7
2	2	0.5	−0.3 or −0.7	−0.3 or −0.7
3	2	−0.5	−0.3 or −0.7	−0.3 or −0.7
4	1	0	−0.3 or −0.5	−0.3 or −0.5
5	1	0.3	−0.3 or −0.5	−0.3 or −0.5
6	1	−0.3	−0.3 or −0.5	−0.3 or −0.5

We consider five tests. The first is the oracle test using the true correlation structure between X^*^ and MEs. When one of two measurements does not have measurement error, the oracle test is the standard t-test based on the correctly measured measurement only. If both measurements are mismeasured, the oracle test uses the optimal weights, *E*[XX^*^] and *E*[ZX^*^], for *X* and *Z*, respectively, to generate the optimal linear combination *W*. Then the oracle test is the standard t-test based on the OLS estimator from a regression of *Y* on *W*. We also consider t-tests based on the OLS estimator from a regression of *Y* on *X* (“OLS w/ x”) and of *Y* on *Z* (“OLS w/ z”), respectively. The fourth (“IV x|z”) is the t-test based on the IV estimator from a regression of *Y* on *X* using *Z* as an instrument. The last (“tmax”) is the maximal t-test with the multiplier bootstrap critical value described in Section 3. We use B=1,000 bootstrap samples and an equally spaced grid of weights in the interval [0, 1] with a distance of 0. 2 between the grid points. All tests are implemented with the homoskedastic variance estimator described in Remark 2 and a nominal level of 0. 05.

**Figure 1.**
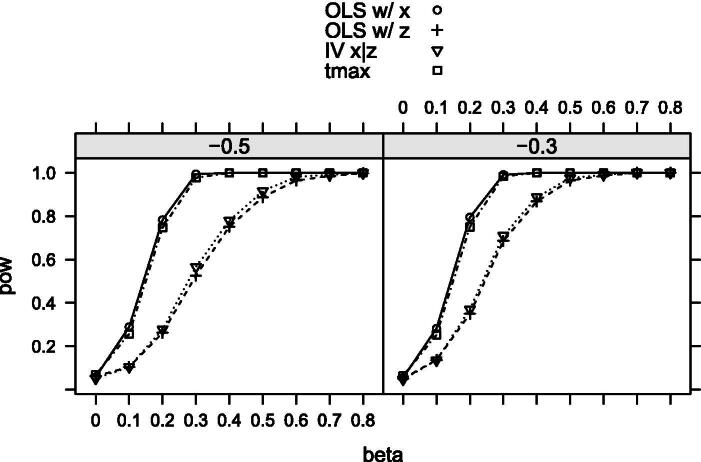
Rejection probabilities in scenario 0, for $\sigma_{X^{*}V}\in \{-0.3,-0.5\}$ varying from left to right.

We first investigate Scenario 0 where *X* does not have measurement error ($X=X^{*}$) and only *Z* is contaminated. Figure 1 shows the power curve for each test. The size is well controlled under the null by all the tests considered. The OLS-based test using *X* only is as powerful as expected. Our maximal t-test performs very similarly to the oracle test, although it is slightly less powerful as we do not use the information on the true correlation structure. The optimal weight is on average very close to 1 whenever β≥0.2, meaning that our test only uses *X* when the null is not true. The other two tests are far less powerful than the oracle test. The same pattern is observed when we adjust the values of $\sigma_{V}^{2}$ and $\sigma_{X^{*}V}.$ This result shows that our test is nearly optimal if one of the two measurements does not suffer from measurement error.

**Figure 2.**
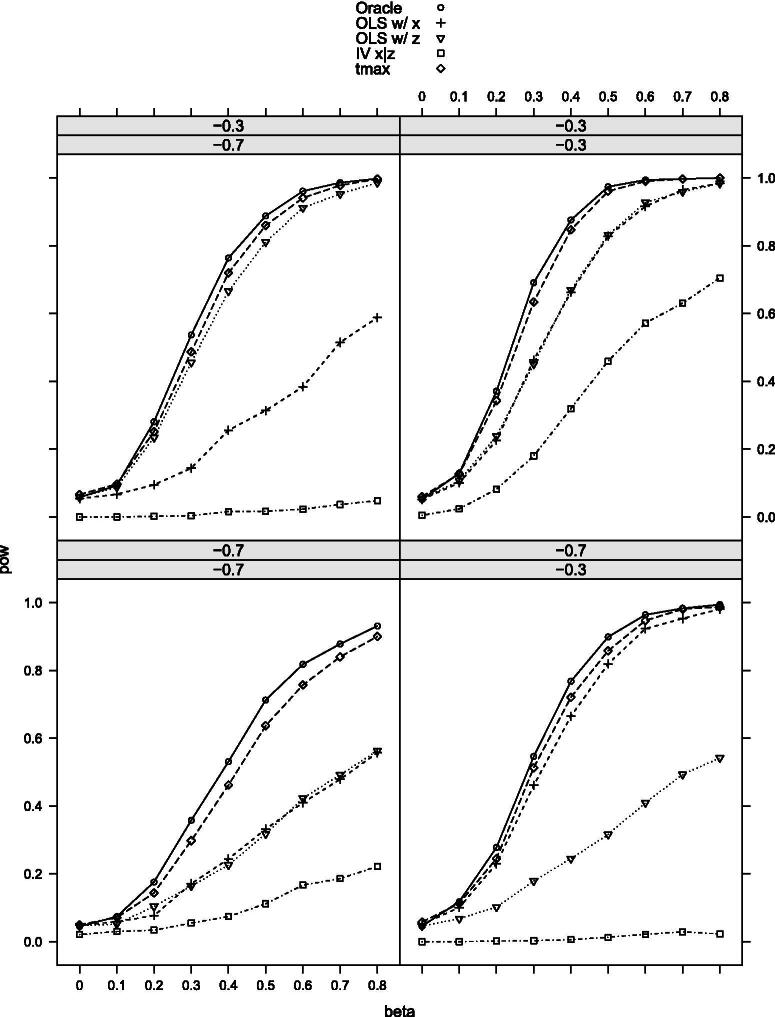
Rejection probabilities in scenario 1, for $\sigma_{X^{*}V}\in \{-0.3,-0.7\}$ varying from top to bottom and $\sigma_{X^{*}U}\in \{-0.3,-0.7\}$ varying from left to right.

**Figure 3.**
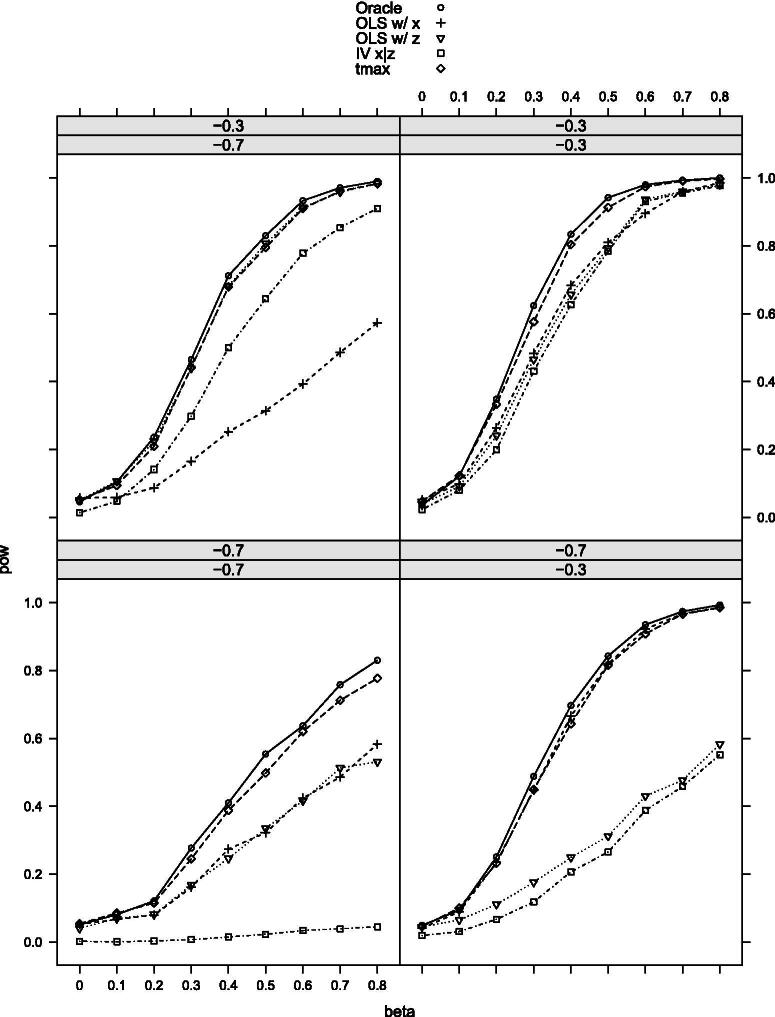
Rejection probabilities in scenario 2, for $\sigma_{X^{*}V}\in \{-0.3,-0.7\}$ varying from top to bottom and $\sigma_{X^{*}U}\in \{-0.3,-0.7\}$ varying from left to right.

**Figure 4.**
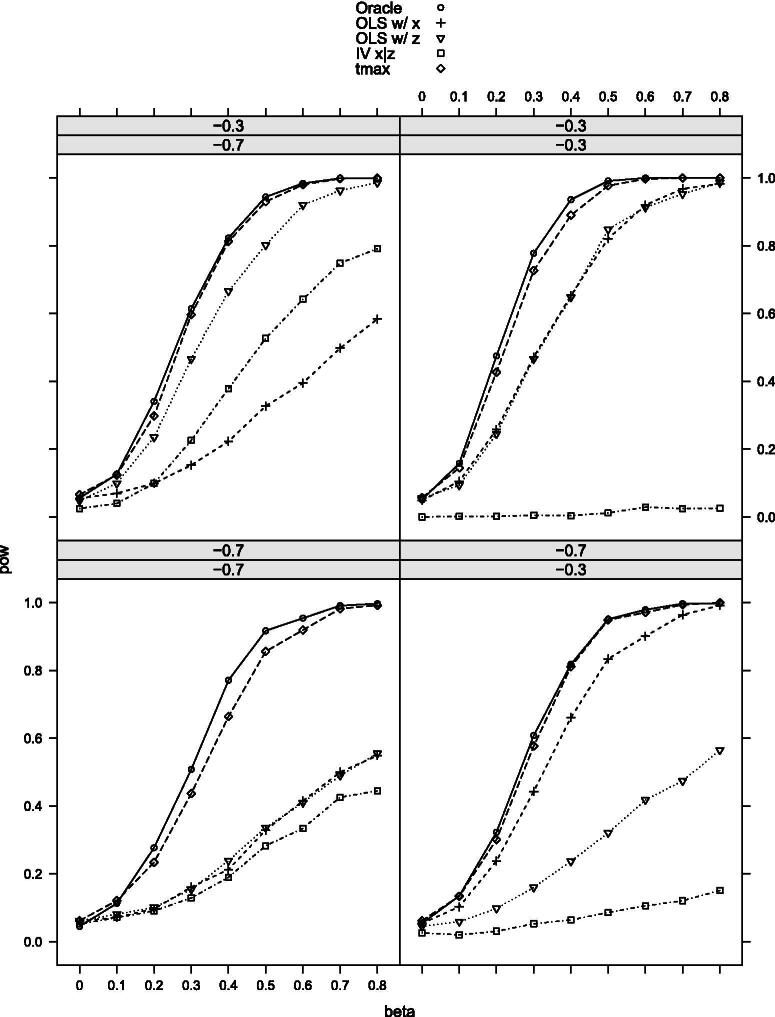
Rejection probabilities in scenario 3, for $\sigma_{X^{*}V}\in \{-0.3,-0.7\}$ varying from top to bottom and $\sigma_{X^{*}U}\in \{-0.3,-0.7\}$ varying from left to right.

**Figure 5.**
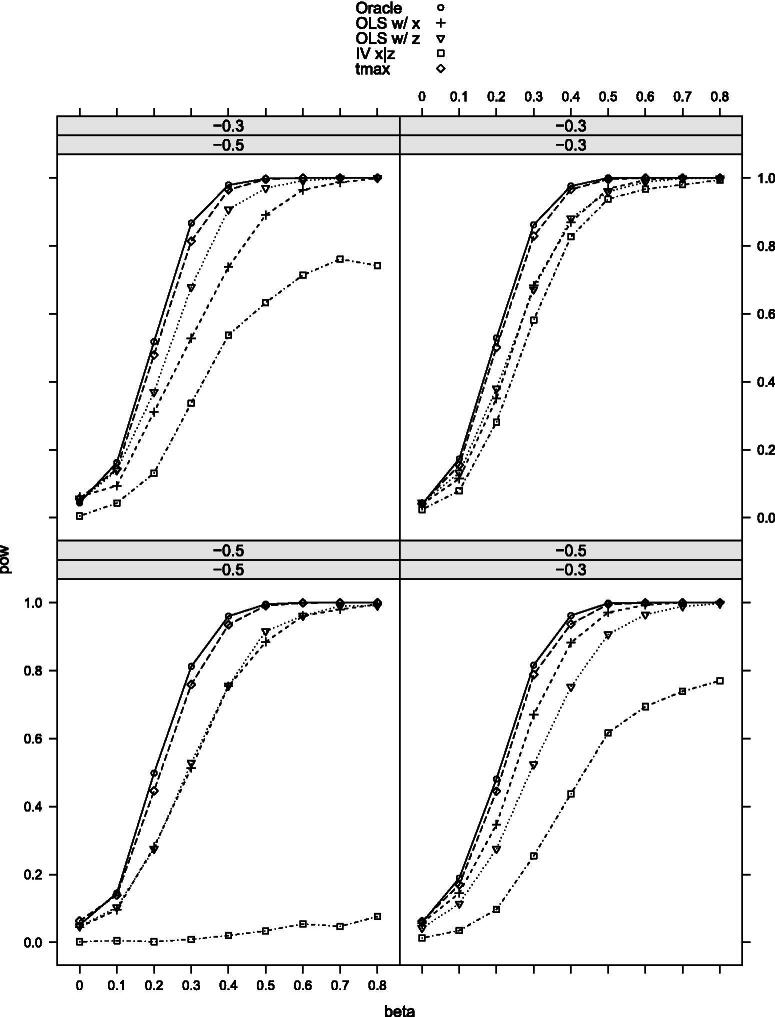
Rejection probabilities in scenario 4, for $\sigma_{X^{*}V}\in \{-0.3,-0.5\}$ varying from top to bottom and $\sigma_{X^{*}U}\in \{-0.3,-0.5\}$ varying from left to right.

**Figure 6.**
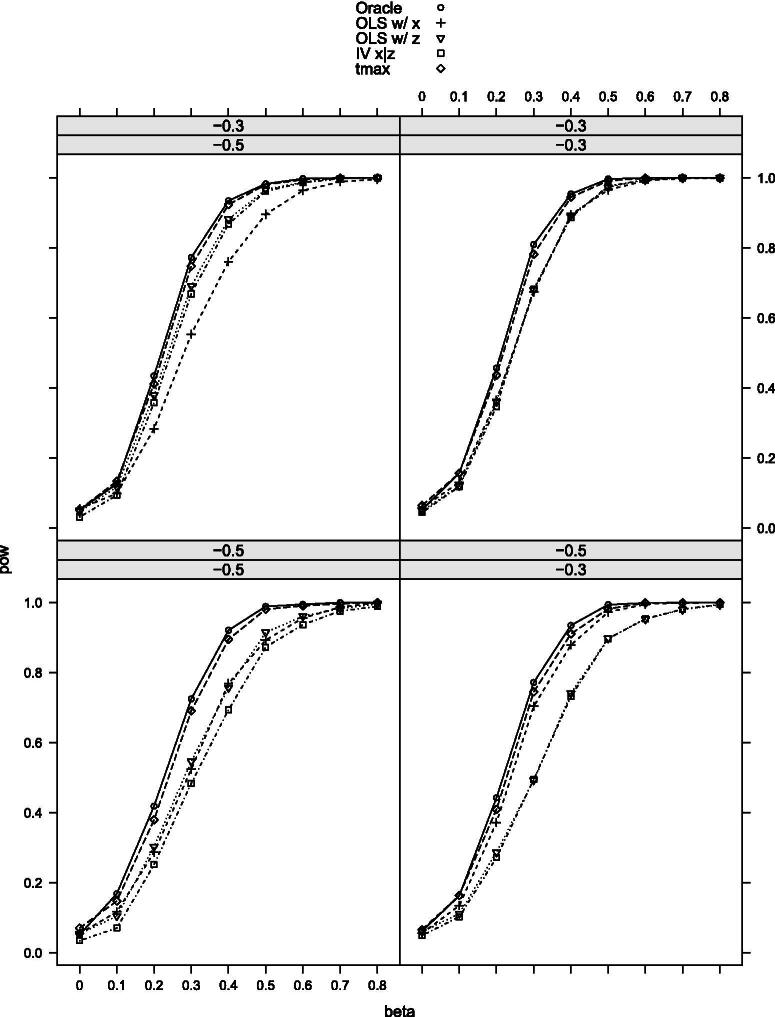
Rejection probabilities in scenario 5, for $\sigma_{X^{*}V}\in \{-0.3,-0.5\}$ varying from top to bottom and $\sigma_{X^{*}U}\in \{-0.3,-0.5\}$ varying from left to right.

**Figure 7.**
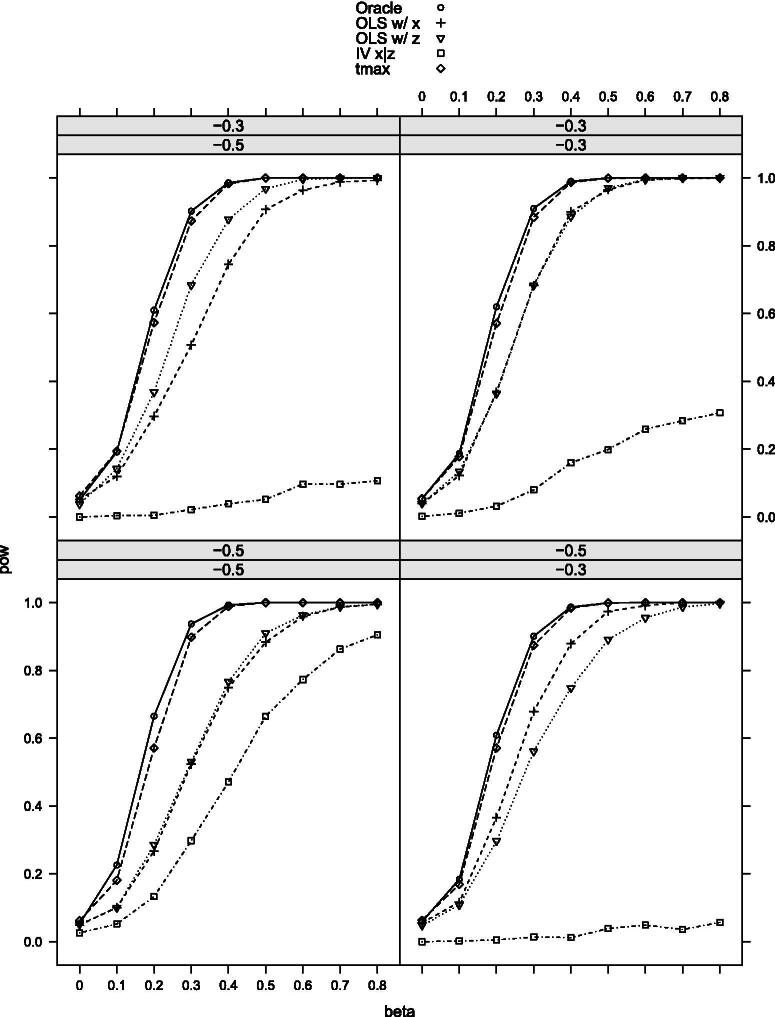
Rejection probabilities in scenario 6, for $\sigma_{X^{*}V}\in \{-0.3,-0.5\}$ varying from top to bottom and $\sigma_{X^{*}U}\in \{-0.3,-0.5\}$ varying from left to right.

Table 2 provides the null rejection frequencies of the tests, and Figs. 2–7 the power curves for scenarios 1–6. All tests control the size well as expected. The power, however, varies significantly among them. The findings can be summarized as follows. First, our maximal t-test performs very similarly to the Oracle test, which is obviously most powerful in all scenarios. Second, in none of the scenarios and parameter combinations, the maximal t-test is dominated by another nonoracle test. In most of them, the maximal t-test strictly dominates all other nonoracle tests (e.g., in scenarios 3 and 6). When the maximal t-test does not strictly dominate all other tests, then it does strictly dominate all but one of the tests, with the latter possessing power roughly equal to that of the maximal t-test. In most of the scenarios and parameter combinations, the power gains of the maximal t-test relative to the others are significant (e.g., scenarios 3 and 6). Third, a common feature among all simulation results is that when the measurement *X* is more precise than *Z*, then the OLS-based test using *X* dominates the OLS-based test using *Z* and the former is closer to the maximal t-test in terms of power (compare, for example, the upper-left and the lower-right panels in Fig. 2). Our maximal t-test either dominates both or is equal to the more powerful of the two tests. In this sense, it automatically adapts to the unknown covariance structure in the measurement system and puts more weight on the more informative measurement. Lastly, in almost all scenarios and parameter combinations, the IV-based t-test performs the poorest.

**Table 2.** Null rejection probabilities in the six different scenarios.

$\sigma_{X^{*}U}$	$\sigma_{X^{*}V}$	Test	S1	S2	S3	S4	S5	S6
strong	strong	Oracle	0.045	0.051	0.045	0.051	0.052	0.053
		OLS w/ x	0.048	0.052	0.060	0.045	0.055	0.049
		OLS w/ z	0.047	0.040	0.062	0.047	0.054	0.051
		IV x|z	0.021	0.002	0.052	0.002	0.035	0.026
		tmax	0.050	0.054	0.062	0.063	0.071	0.062
strong	weak	Oracle	0.058	0.046	0.056	0.044	0.052	0.050
		OLS w/ x	0.054	0.057	0.055	0.062	0.056	0.052
		OLS w/ z	0.058	0.050	0.048	0.049	0.047	0.038
		IV x|z	0.000	0.014	0.025	0.005	0.032	0.000
		tmax	0.066	0.052	0.066	0.056	0.053	0.062
weak	strong	Oracle	0.046	0.049	0.054	0.058	0.062	0.059
		OLS w/ x	0.051	0.041	0.054	0.057	0.056	0.056
		OLS w/ z	0.046	0.045	0.045	0.042	0.060	0.048
		IV x|z	0.000	0.019	0.026	0.013	0.050	0.000
		tmax	0.058	0.046	0.062	0.062	0.066	0.063
weak	weak	Oracle	0.052	0.036	0.051	0.039	0.053	0.054
		OLS w/ x	0.052	0.052	0.049	0.040	0.058	0.040
		OLS w/ z	0.053	0.039	0.055	0.044	0.050	0.040
		IV x|z	0.005	0.023	0.000	0.024	0.045	0.002
		tmax	0.060	0.047	0.057	0.041	0.065	0.054

The values of $\sigma_{X^{*}U}$ and $\sigma_{X^{*}V}$ vary between “strong” (-0.7 for scenarios 1–3; -0.5 for scenarios 4–6) and “weak” (−0.3 for all scenarios)

Exogeneity of X^*^ and nondifferentiability of MEs are crucial for the transformed null (4) being an observable implication of β=0. In Appendix C, we show in further simulations that our tests are robust to *small* deviations from nondifferentiability, at least for the data-generating processes we consider. This robustness property may, of course, be lost for other data-generating processes.

## 5. Empirical illustration: returns to schooling

In this section, we revisit the empirical work by Bonjour et al. (2003) who studied the returns to schooling using data from monozygotic twins.^3^ Bonjour et al. (2003) estimate that each additional year of schooling increases hourly wage by 7.7%, which is statistically significant at the 5% level.^4^ Amin (2011) argues that this result is largely driven by a few outliers in the data set. In particular, one pair of twins has a very large difference in their hourly earnings (£94.18) with only a 2-year schooling gap, whereas the average difference of hourly wages with a 2-year difference in education is only £7.74. Amin (2011) shows that, after removing this outlier, the estimated return to schooling is only 5.1% and it is no longer significant at the 5% level.

This section aims to apply our new t-test, which in simulations is more powerful than standard OLS- and IV-based t-tests, and see whether we can find statistically significant returns to schooling in the UK data set. To this end, for a pair of twins, let *Y*_1_ and *Y*_2_ be log wages of the first and second twins. Denote by *X*_1_ and *X*_2_ their respective self-reported levels of education. In addition, the second twin is asked to report the education of the first twin, *Z*_1_, and the first to report the education of the second, *Z*_2_. We consider the regression models (1) and (6) with $Y=Y_{1}-Y_{2}$ the difference of log wages, X^*^ the difference of true education levels, $X=X_{1}-X_{2}$ the difference of self-reported education, and $Z=Z_{1}-Z_{2}$ the difference of cross-reported education. *R* contains additional covariates such as marital status and work experience. To study the returns to schooling in the UK, we use Bonjour et al.’s (2003) data set, which is publicly available.

First, we test the null of no ME in self-reported education levels using the nonparametric test in Wilhelm (2019).^5^ The test statistic is 0. 00115 and the critical value 0. 00361, leading to a p-value of 0. 4. Hence, we cannot reject the null hypothesis of no ME in self-reported education. There is not enough information in the data set to reject the null. This could simply be because the sample size (214) is too small, so the nonparametric test does not have enough power. Therefore, in the second step, we impose the linearity of the wage equation and perform the test of no ME again. To do this, we regress *Y* onto both *X* and *Z* (and a constant). We find that the p-value of the coefficient estimate of *Z* is around 0. 1 (t-statistic of 1. 628). Therefore, with the assumption of linearity, we still suspect the presence of measurement errors, while the tests do not strongly reject the null of no ME. (Hausman, 1978; Lee and Wilhelm, 2020). This finding is consistent with the possibility that the nonparametric test has low power because of the small sample size and thus fails to reject, but the test imposing the linearity of the conditional expectation is more powerful. Since the education variable is discrete, we know that, by construction, ME (if there is any) cannot be classical because it has to depend on the true level of education.

We now apply our maximal t-test imposing homoskedasticity (both Bonjour et al. (2003) and Amin (2011) assume homoskedasticity, too) as described in Remark 2. We implement our maximal t-test with B=5,000 bootstrap replications and an equally-spaced grid of weights $\omega_{1},\ldots ,\omega_{p}$ such that $\omega_{j}=((j-1)/n,1-(j-1)/n,(j-1)/n,1-(j-1)/n)$ for j=1,…,p=n+1. We have also used grids that are twice the size as well as half the size, but the test results were almost identical. We consider several subsamples of the data, using from 30 to 214 (the full sample) observations. Table 3 shows the results of our maximal t-test (“maxt”) and compares it to three other tests: the t-test based on the OLS estimator from a regression of *Y* on *X* (“OLS w/ x”), the t-test based on the OLS estimator from a regression of *Y* on *Z* (“OLS w/ z”), and the t-test based on the IV estimator from a regression of *Y* on *X* using *Z* as instrument (“IV”). The latter three tests also employ variance estimators imposing homoskedasticity. The table also shows the maximizer of our t-statistic (“${\hat{a}}^{*}$”) defined as the first element of the grid point *ω*_*j*_ at which the t-statistic is maximized, the 95%-critical value (“95% CV”), and the *p*-value of our test from the multiplier bootstrap.

**Table 3.** Tests using homoskedastic standard errors applied to first *n* observations of the data set from Bonjour et al. (2003); rejections at the 5% level are highlighted in bold.

n	OLS w/ x	OLS w/ z	IV	${\hat{a}}^{*}$	tmax	95% CV	p-value
30	0.35	0.22	0.22	0.77	0.36	1.41	0.77
40	0.26	0.24	0.24	0.55	0.30	1.24	0.79
50	0.86	0.77	0.77	0.58	0.94	1.26	0.16
60	1.00	0.94	0.94	0.57	1.08	1.26	0.10
70	1.17	1.32	1.31	0.36	**1.38**	1.17	0.02
80	1.04	1.35	1.33	0.21	**1.37**	1.16	0.02
90	1.22	1.24	1.24	0.47	**1.37**	1.21	0.02
100	1.27	1.23	1.23	0.55	**1.36**	1.18	0.02
110	**1.96**	**2.61**	**2.55**	0.04	**2.62**	2.20	0.02
120	**1.99**	**2.76**	**2.69**	0.00	**2.76**	2.25	0.02
130	**2.01**	**3.31**	**3.16**	0.00	**3.31**	2.28	0.00
140	**1.96**	**3.19**	**3.06**	0.00	**3.19**	2.22	0.01
150	**2.16**	**3.05**	**2.97**	0.00	**3.05**	2.20	0.01
160	**2.24**	**2.95**	**2.89**	0.09	**2.96**	2.27	0.01
170	**2.04**	**3.19**	**3.07**	0.02	**3.20**	2.18	0.00
180	1.64	**2.79**	**2.70**	0.00	**2.79**	2.25	0.02
190	**2.02**	**2.92**	**2.85**	0.06	**2.92**	2.14	0.01
200	**1.97**	**2.73**	**2.68**	0.09	**2.73**	2.28	0.01
214	1.74	**2.37**	**2.34**	0.10	**2.38**	2.22	0.04

Our maximal t-test rejects the null of no effect for all sample sizes greater than 60. Bonjour et al. (2003) consider the OLS estimator using *X* and the IV estimator using *Z* as instrument for *X*, among others. These do reject the full sample, but not all smaller samples. The OLS-based and IV-based tests reject the null for 110 or larger sizes. The findings in this empirical application are consistent with the simulation results. They could, for example, be interpreted similarly to the upper-right panel of Figs. 3 and 6. It is plausible that twins may have similar “ability to report correctly” in the sense that *U* and *V* are positively correlated. As the OLS-based test using *Z* does not reject more often than the one using *X*, the findings are consistent with *X* and *Z* being similarly mismeasured. The covariance structure of $\left(X^{*},U,V\right)$ is not identified and thus cannot be consistently estimated, so we cannot learn from data a priori which measurement is more precise, whether linear combinations of measurements help improve power, and therefore which of the many different possible OLS and IV tests to use. However, our maximal t-test adapts to the unknown covariance structure, thus does not require such knowledge, and is more powerful. Without assuming homoskedasticity, all tests perform similarly and start rejecting around sample sizes of 50−70 as reported in Table 4.^6^

**Table 4.** Tests using heteroskedasticity and robust standard errors applied to first *n* observations of the data set from Bonjour et al. (2003); rejections at the 5% level are highlighted in bold.

n	OLS w/ x	OLS w/ z	IV	${\hat{a}}^{*}$	tmax	95% CV	p-value
30	0.49	0.46	0.48	0.43	0.56	2.25	0.81
40	0.43	0.59	0.61	0.20	0.61	2.23	0.77
50	1.34	1.77	**2.00**	0.28	1.93	2.27	0.11
60	1.58	**2.16**	**2.39**	0.27	**2.31**	2.23	0.04
70	**1.98**	**3.13**	**3.16**	0.23	**3.30**	2.28	0.00
80	1.78	**3.21**	**3.28**	0.18	**3.31**	2.29	0.00
90	**2.03**	**2.83**	**2.96**	0.26	**3.09**	2.28	0.01
100	**2.22**	**2.73**	**2.82**	0.29	**2.94**	2.29	0.01
110	**2.80**	**2.30**	**2.16**	0.94	**2.81**	2.24	0.01
120	**2.80**	**2.38**	**2.24**	0.87	**2.83**	2.18	0.01
130	**3.03**	**2.86**	**2.57**	0.75	**3.17**	2.23	0.00
140	**2.92**	**2.80**	**2.52**	0.72	**3.08**	2.22	0.00
150	**2.90**	**2.67**	**2.43**	0.73	**3.04**	2.24	0.01
160	**3.01**	**2.56**	**2.35**	0.82	**3.08**	2.26	0.00
170	**2.76**	**2.90**	**2.48**	0.60	**3.07**	2.27	0.01
180	**2.11**	**2.46**	**2.18**	0.34	**2.50**	2.19	0.02
190	**2.59**	**2.65**	**2.36**	0.60	**2.85**	2.22	0.01
200	**2.49**	**2.42**	**2.22**	0.67	**2.67**	2.22	0.02
214	**2.20**	**2.12**	**1.99**	0.67	**2.35**	2.22	0.04

We also test the null hypothesis of no returns to schooling after removing outliers. As Amin (2011) suggests, we first drop an observation that shows the absolute difference in hourly wages > 90. Then, we sequentially drop further outliers with the absolute difference greater than 75, 65, and 60. In each step, we lose 1 observation. The results are displayed in Table 5. After dispensing with the most extreme outlier, the OLS and IV estimates are no longer significant, even at the 10% level. Our maximal t-test, on the contrary, rejects the null hypothesis of no effect at around the 6.5% level. The maximal t-test results remain significant at the 5.6% and 7.7% levels after we drop the second and third most extreme outliers, respectively. And thereby, our test supports the original conclusion in Bonjour et al. (2003) that the returns to schooling are significantly different from 0 even when a few extreme outliers are removed from the sample.

**Table 5.** Robustness of the results when outliers are removed; the numbers in parentheses are *p*-values.

	OLS	IV	${\hat{a}}^{*}$	tmax
Full sample	1. 744	2. 340	0. 10	2. 376
(*n* = 214)	(0. 083)	(0. 020)		(0. 034)
abs. wage dif. < 90	1. 495	1. 616	0. 39	1. 705
(*n* = 213)	(0. 136)	(0. 108)		(0. 065)
abs. wage dif. < 75	1. 528	1. 651	0. 39	1. 742
(*n* = 212)	(0. 128)	(0. 100)		(0. 056)
abs. wage dif. < 65	1. 572	1. 228	0. 84	1. 584
(*n* = 211)	(0. 118)	(0. 221)		(0. 077)
abs. wage dif. < 60	1. 496	1. 313	0. 68	1. 545
(*n* = 210)	(0. 136)	(0. 191)		(0. 114)

As a final robustness check, we also include additional covariates in the regression. The dataset contains various characteristics of twins. As in Bonjour et al. (2003) and Amin (2011), we control for marital status, working part-time, region to live, and current work experience; see Table 6. The inclusion of these covariates further decreases our maximal t-test’s *p*-values so that it rejects the null at the 5% level even when all four most extreme observations are removed. The OLS and IV estimates, on the other hand, are only significant in 1 or 2 subsamples at the 10% level.

**Table 6.** Robustness of the results with additional covariates (marriage, working part-time, living in London or the South East, and work experience) when outliers are removed; the numbers in parentheses are *p*-values.

	OLS	IV	${\hat{a}}^{*}$	tmax
Full sample	1. 628	2. 312	0. 02	2. 350
(*n* = 214)	(0. 105)	(0. 022)		(0. 037)
abs. wage dif. < 90	1. 495	1. 630	0. 38	1. 714
(*n* = 213)	(0. 137)	(0. 105)		(0. 065)
abs. wage dif. < 75	1. 622	1. 733	0. 41	1. 837
(*n* = 212)	(0. 107)	(0. 085)		(0. 050)
abs. wage dif. < 65	1. 715	1. 288	0. 90	1. 719
(*n* = 211)	(0. 088)	(0. 199)		(0. 047)
abs. wage dif. < 60	1. 833	1. 510	0. 77	1. 861
(*n* = 210)	(0. 068)	(0. 133)		(0. 047)

In conclusion, employing our maximal t-test, which remains powerful even in the presence of nonclassical ME in schooling, we have been able to recover statistically significant returns to schooling even after removing the most extreme observations. This finding is in contrast to those in Amin (2011) and provides further robustness in support of the original findings by Bonjour et al. (2003). This empirical application clearly illustrates that our new test can secure the statistical significance of estimates of interest when standard t-tests cannot.

## Figures and Tables

**Figure B1.**
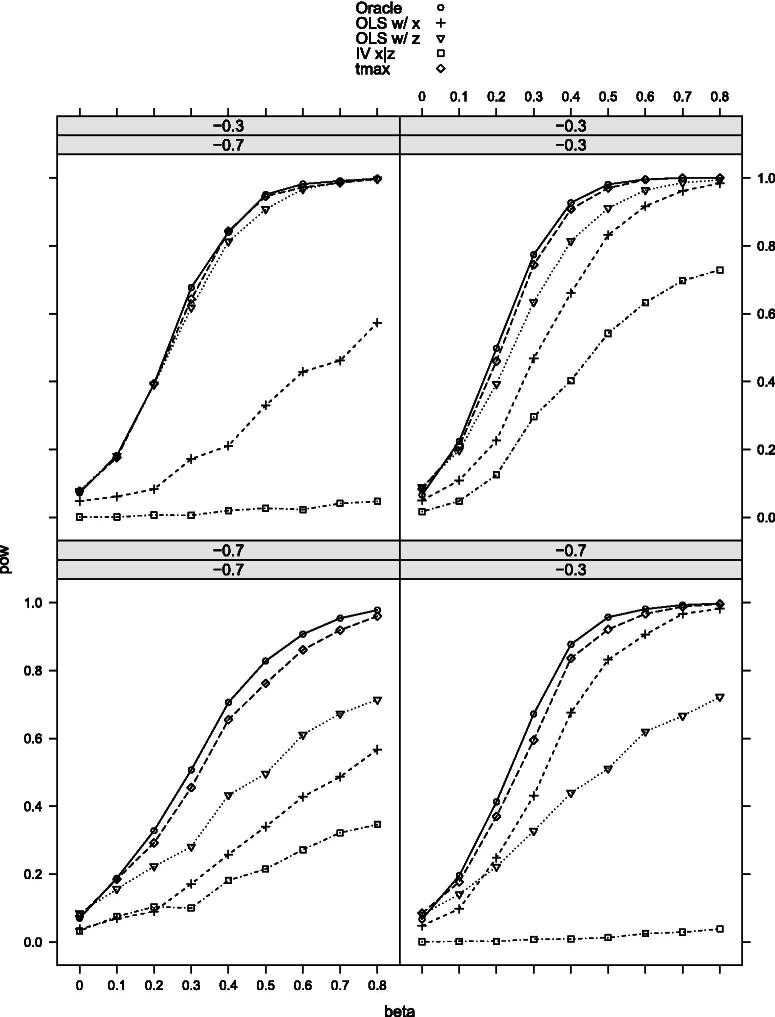
Rejection probabilities in scenario 1, for $\sigma_{X^{*}V}\in \{-0.3,-0.7\}$ varying from top to bottom and $\sigma_{X^{*}U}\in \{-0.3,-0.7\}$ varying from left to right.

**Figure B2.**
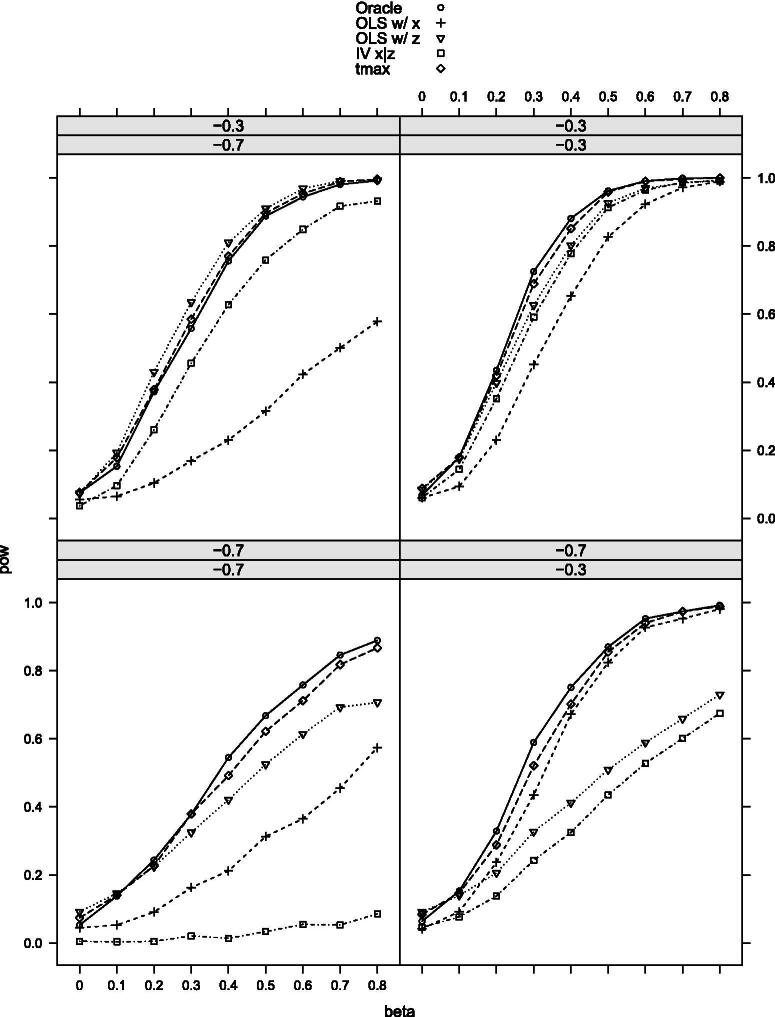
Rejection probabilities in scenario 2, for $\sigma_{X^{*}V}\in \{-0.3,-0.7\}$ varying from top to bottom and $\sigma_{X^{*}U}\in \{-0.3,-0.7\}$ varying from left to right.

**Figure B3.**
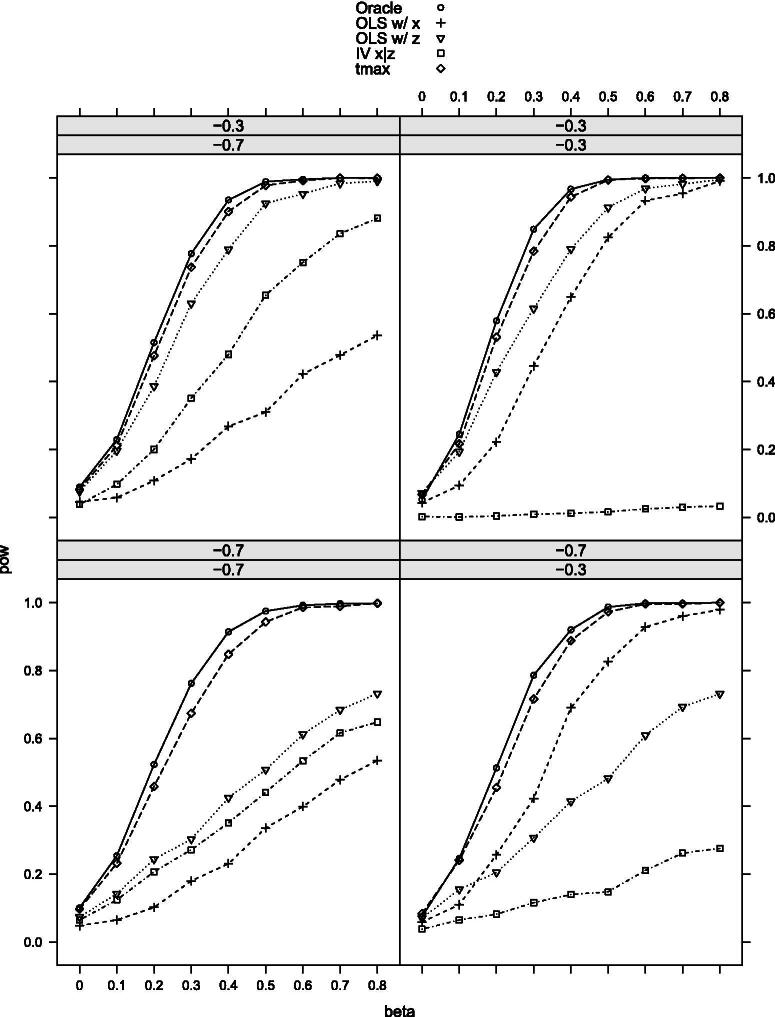
Rejection probabilities in scenario 3, for $\sigma_{X^{*}V}\in \{-0.3,-0.7\}$ varying from top to bottom and $\sigma_{X^{*}U}\in \{-0.3,-0.7\}$ varying from left to right.

**Figure B4.**
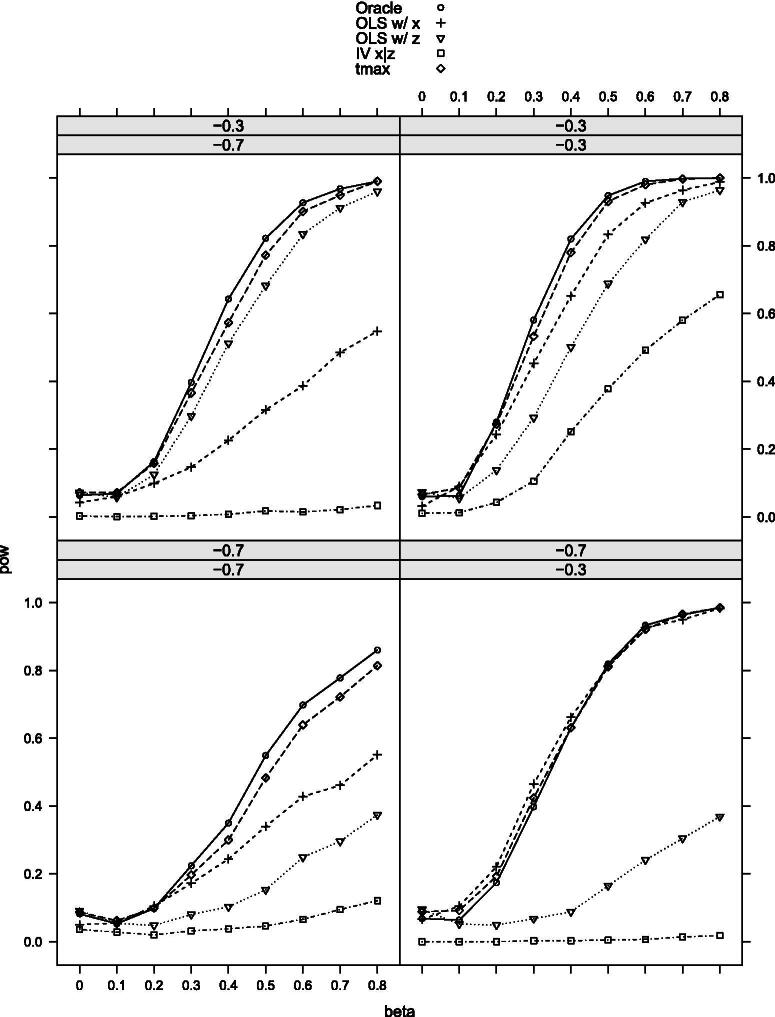
Rejection probabilities in scenario 4, for $\sigma_{X^{*}V}\in \{-0.3,-0.7\}$ varying from top to bottom and $\sigma_{X^{*}U}\in \{-0.3,-0.7\}$ varying from left to right.

**Figure B5.**
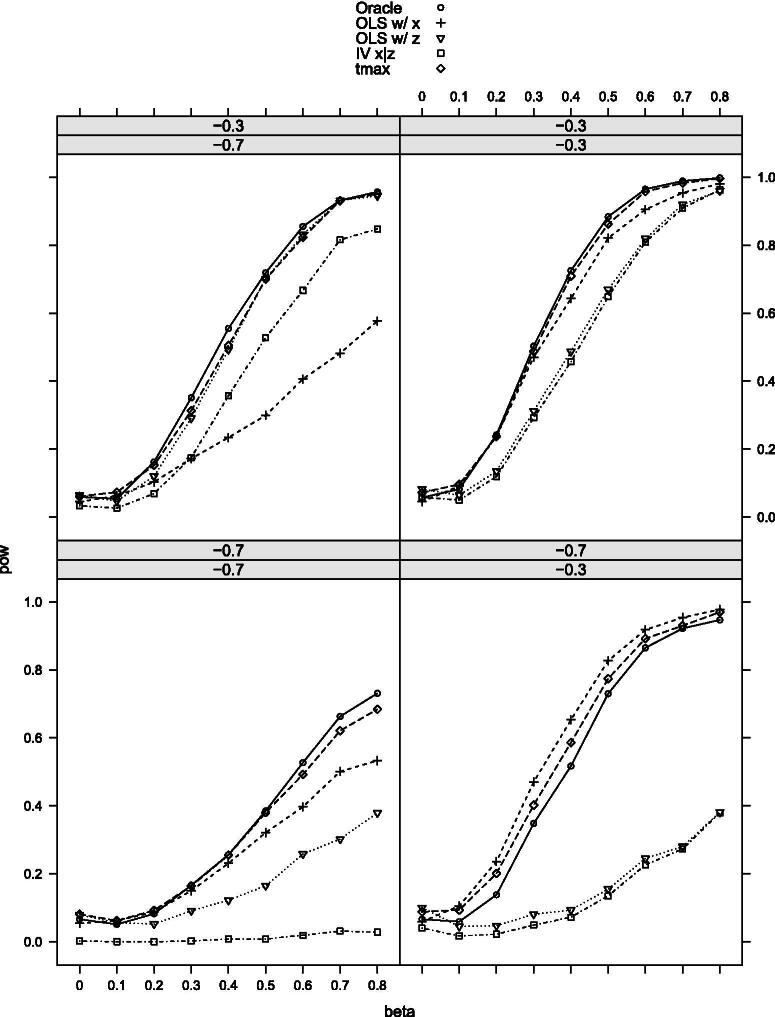
Rejection probabilities in scenario 5, for $\sigma_{X^{*}V}\in \{-0.3,-0.7\}$ varying from top to bottom and $\sigma_{X^{*}U}\in \{-0.3,-0.7\}$ varying from left to right.

**Figure B6.**
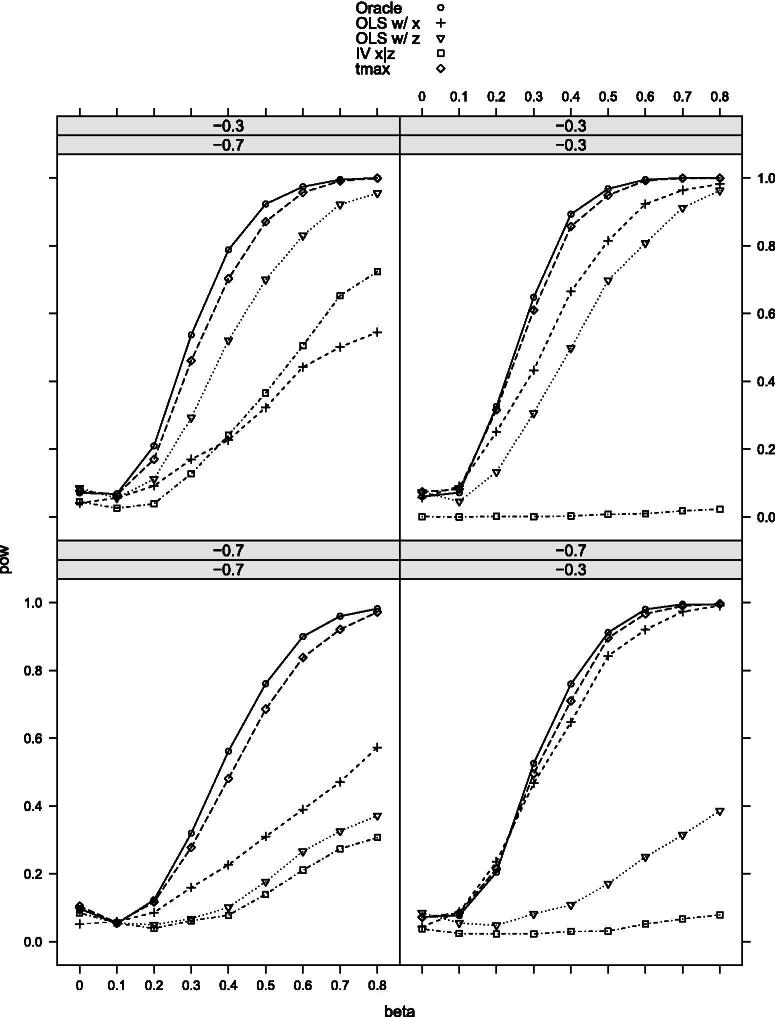
Rejection probabilities in scenario 6, for $\sigma_{X^{*}V}\in \{-0.3,-0.7\}$ varying from top to bottom and $\sigma_{X^{*}U}\in \{-0.3,-0.7\}$ varying from left to right.

## Notes

1. Given exogeneity of *X*^*^, the nondifferentiability of measurement errors is crucial for $\beta_{OLS}=\beta_{IV}=0$ being an observable implication of the null.

2. Lemma 2 in Appendix B provides a slightly modified statement and derivation for those bounds.

3. The UK twins data were collected from St. Thomas’ U.K. Adult Twin Registry.

4. Ashenfelter and Krueger (1994) estimated a much larger return (1–16%, significant at the 1% level) using the US twins data. Rouse (1999) extended the US data set by adding survey results from subsequent years (1992, 1993, and 1995) and suggesting that the returns may be much smaller (9.5%). She also found mixed evidence of their statistical significance.

5. We implement the test based on Delgado and Manteiga’s (2001) Cramér-von Mises statistic for the null hypothesis (9) in Wilhelm (2019) with *μ*(*y*) = *y*, a Gaussian kernel, and 1, 000 bootstrap replications.

6. The OLS and IV t-tests are implemented with Eicker-Huber-White heteroskedasticity and robust standard errors.

## Appendix A finiteness of the optimal weights

Assumption 7.
*(i) *E*[*X**Y*]≠0, *E*[*Z**Y*]≠0, and (ii) $|\text{corr}(X^{*},(Z-X))|\neq 1$.*

The possibility of $|a^{*}|=\infty$ can be ruled out under a set of weak assumptions. The first part implies that *β*≠0. It also rules out the cases in which the MEs are perfectly negatively correlated with the true variable ($E[(X^{*}{)}^{2}]=-E[X^{*}U]=-E[X^{*}V]$). The last part means that the true variable is not recovered from *Z*−*X*.

Lemma 1. *Suppose (1) and Assumptions 1 and 7 hold. Then, the maximal weight *a*^*^ is finite.*

$$\begin{array}{l} |a^{*}|<\infty \end{array}$$

Proof of Lemma 1. The numerator of *a*^*^ in Theorem 1 is finite if the denominator is nonzero, then $|a^{*}|<\infty$.

$$\begin{array}{l} C:=E[X(Z-X)]E[ZY]-E[Z(Z-X)]E[XY] \end{array}$$

By Assumption 1 (i), $E[XY]=\beta E[XX^{*}]$ and $E[ZY]=\beta E[ZX^{*}]$. Then *C* trivially becomes zero when *β* = 0 or $E[(X^{*}{)}^{2}]+E[X^{*}U]=E[(X^{*}{)}^{2}]+E[X^{*}V]=0$. These are ruled out by Assumption 7. The other cases in which *C* is zero are the following.

1. E[Z(Z−X)]=0 and E[X(Z−X)]=0
2. $\frac{E[X(Z-X)]}{E[Z(Z-X)]}=\frac{E[XY]}{E[ZY]}=\frac{E[(X^{*}{)}^{2}]+E[X^{*}U]}{E[(X^{*}{)}^{2}]+E[X^{*}V]}$

The first case implies that *X* = *Z* and is ruled out by (iii) of Assumption 1. The second case indicates that the ratio of *E*[*X*(*Z*−*X*)] to *E*[*Z*(*Z*−*X*)] is equal to the ratio of $E[(X^{*}{)}^{2}]+E[X^{*}U]$ to $E[(X^{*}{)}^{2}]+E[X^{*}V]$. Therefore, for a constant *γ*≠0,

$$\begin{array}{l} E[XZ-X^{2}]=\gamma (E[(X^{*}{)}^{2}]+E[X^{*}U]),\;E[Z^{2}-XZ]=\gamma (E[(X^{*}{)}^{2}]+E[X^{*}V]). \end{array}$$

By simple algebra,

$$\begin{array}{l} E[X^{*}V]-(1+\gamma )E[X^{*}U]+E[UV]-E[U^{2}]=\gamma E[(X^{*}{)}^{2}] \end{array}$$

$$\begin{array}{l} \left(1-\gamma )E[X^{*}V]-E[X^{*}U]+E[V^{2}]-E[UV]=\gamma E[(X^{*}{)}^{2}\right] \end{array}$$

and therefore,

$$\begin{array}{l} \gamma (E[X^{*}V]-E[X^{*}U])=E[V^{2}]-2E[UV]+E[U^{2}] \end{array}$$

and this implies that

$$\begin{array}{l} \gamma E[X^{*}(V-U)]=E[(V-U{)}^{2}]\;⟹X^{*}=\frac{1}{\gamma }(V-U)=\frac{1}{\gamma }(Z-X). \end{array}$$

As Assumption 7 rules out perfect positive and negative correlation between *X*^*^ and *Z*−*X*, such a possibility is ruled out. Therefore, the denominator *C* is nonzero, and hence *a*^*^ is finite. ▪

## Appendix B Proofs

### B.1. Proofs for Section 2

Proof of Theorem 1. First, we show that

(B1) $$\begin{array}{l} \underset{\omega \in R^{4}}{\sup}t(\omega {)}^{2}=\underset{a\in R}{\sup}t(a,1-a,a,1-a{)}^{2}. \end{array}$$

To this end, notice that given $\omega =(\omega_{1},\omega_{2},\omega_{3},\omega_{4}),$

$$\begin{array}{l} \beta (\omega )=\frac{E[W(\omega_{1},\omega_{2})Y]}{E[W(\omega_{1},\omega_{2})W(\omega_{3},\omega_{4})]},\;\sigma^{2}(\omega )=\frac{\sigma_{\varepsilon }^{2}(\omega )E\left[W(\omega_{1},\omega_{2}{)}^{2}\right]}{(E\left[W(\omega_{1},\omega_{2})W(\omega_{3},\omega_{4})\right]{)}^{2}} \end{array}$$

and the t-ratio becomes

$$\begin{array}{l} t(\omega )=\frac{\beta (\omega )}{\sigma (\omega )}=\frac{E[W(\omega_{1},\omega_{2})Y]}{\sigma_{\varepsilon }(\omega )\sqrt{E\left[W(\omega_{1},\omega_{2}{)}^{2}\right]}}. \end{array}$$

$\sigma_{\varepsilon }^{2}(\omega )=E[(Y-\beta (\omega )W(\omega_{3},\omega_{4}){)}^{2}]$ is minimized at $\omega =(\omega_{3},\omega_{4},\omega_{3},\omega_{4})$ since the OLS estimand $\beta (\omega_{3},\omega_{4},\omega_{3},\omega_{4})=E[W(\omega_{3},\omega_{4})Y]/E[W(\omega_{3},\omega_{4}{)}^{2}]$ minimizes the residual variance by construction. This implies that

(B2) $$\begin{array}{l} \sigma_{\varepsilon }(\omega_{3},\omega_{4},\omega_{3},\omega_{4})\leq \sigma_{\varepsilon }(\omega_{1},\omega_{2},\omega_{3},\omega_{4}). \end{array}$$

Let $\left(\omega_{3}^{*},\omega_{4}^{*}\right)$ be the minimizer of $\sigma_{\varepsilon }^{2}(\omega_{3},\omega_{4},\omega_{3},\omega_{4}).$ By straightforward algebra,

$$\begin{array}{ll} \sigma_{\varepsilon }^{2}(\omega_{3},\omega_{4},\omega_{3},\omega_{4}) & =E\left[(Y-\frac{E[W(\omega_{3},\omega_{4})Y]}{E[W(\omega_{3},\omega_{4}{)}^{2}]}W(\omega_{3},\omega_{4}){)}^{2}\right]\\ & =E[Y^{2}]-2\frac{E[(W(\omega_{3},\omega_{4})Y]{)}^{2}}{E[W(\omega_{3},\omega_{4}{)}^{2}]}+\frac{{\left(E[W(\omega_{3},\omega_{4})Y]\right)}^{2}}{E[W(\omega_{3},\omega_{4}{)}^{2}]}\\ & =E[Y^{2}]-\frac{E[(W(\omega_{3},\omega_{4})Y]{)}^{2}}{E[W(\omega_{3},\omega_{4}{)}^{2}]}, \end{array}$$

and $\frac{E[(W(\omega_{3},\omega_{4})Y]{)}^{2}}{E[W(\omega_{3},\omega_{4}{)}^{2}]}$ is maximized at $(\omega_{3}^{*},\omega_{4}^{*}).$ This result with (B2) implies that

$$\begin{array}{l} \left|t(\omega )|\leq \frac{1}{\sigma_{\varepsilon }(\omega_{3}^{*},\omega_{4}^{*},\omega_{3}^{*},\omega_{4}^{*})}\left|\frac{E[W(\omega_{3}^{*},\omega_{4}^{*})Y]}{\sqrt{E\left[W(\omega_{3}^{*},\omega_{4}^{*}{)}^{2}\right]}}\right|=|t(\omega_{3}^{*},\omega_{4}^{*},\omega_{3}^{*},\omega_{4}^{*})\right| \end{array}$$

as both $\frac{1}{\sigma_{\varepsilon }(a,b,a,b)}$ and $\left|\frac{E[W(a,b)Y]}{\sqrt{E\left[W(a,b{)}^{2}\right]}}\right|$ are maximized at $(a,b)=(\omega_{3}^{*},\omega_{4}^{*}).$ By Assumption 1(ii), there is no linear combination of *X* and *Z* that is equal to zero so that, together with the positive variance Assumption 1(i), we have $E[W(a,b{)}^{2}]\neq 0$ for all $\left(a,b)\in R^{2}\setminus \{(0,0)\right\}$. Hence, the solution exists. Now we can optimize the t-ratio over only two weights so that

$$\begin{array}{l} \underset{\omega \in R^{4}}{\sup}t^{2}(\omega_{1},\omega_{2},\omega_{3},\omega_{4})=\underset{(\omega_{1},\omega_{2})\in R^{2}}{\sup}t^{2}(\omega_{1},\omega_{2},\omega_{1},\omega_{2})\;\forall \omega \in R^{4}. \end{array}$$

Next, notice that

$$\begin{array}{l} \beta \left(\frac{\omega_{1}}{\omega_{1}+\omega_{2}},\frac{\omega_{2}}{\omega_{1}+\omega_{2}},\frac{\omega_{1}}{\omega_{1}+\omega_{2}},\frac{\omega_{2}}{\omega_{1}+\omega_{2}}\right)=\beta (\omega_{1},\omega_{2},\omega_{1},\omega_{2})(\omega_{1}+\omega_{2}) \end{array}$$

and

$$\begin{array}{l} \sigma^{2}\left(\frac{\omega_{1}}{\omega_{1}+\omega_{2}},\frac{\omega_{2}}{\omega_{1}+\omega_{2}},\frac{\omega_{1}}{\omega_{1}+\omega_{2}},\frac{\omega_{2}}{\omega_{1}+\omega_{2}}\right)=\sigma^{2}(\omega_{1},\omega_{2},\omega_{1},\omega_{2})(\omega_{1}+\omega_{2}{)}^{2}. \end{array}$$

Therefore, the weights in the t-ratio can be standardized to sum up to one,

$$\begin{array}{l} t^{2}\left(\frac{\omega_{1}}{\omega_{1}+\omega_{2}},\frac{\omega_{2}}{\omega_{1}+\omega_{2}},\frac{\omega_{1}}{\omega_{1}+\omega_{2}},\frac{\omega_{2}}{\omega_{1}+\omega_{2}}\right)=t^{2}(\omega_{1},\omega_{2},\omega_{1},\omega_{2}), \end{array}$$

and (B1) follows. By a slight abuse of notation, denote t(a):=t(a,1−a,a,1−a). *t*(*a*) is a twice continuously differentiable function. To find the maximizer of *t*(*a*)^2^, we consider first the first-order condition with respect to *a*,

(B3) $$\begin{array}{l} \frac{\partial }{\partial a}t(a{)}^{2}=0. \end{array}$$

Suppose *C*≠0, then this equation has two solutions,

$$\begin{array}{l} a_{1}^{*}:=\frac{E[YZ]}{E[YZ]-E[XZ]} \end{array}$$

and

$$\begin{array}{l} a_{2}^{*}:=\frac{E[XZ]E[ZY]-E[Z^{2}]E[XY]}{C}. \end{array}$$

The first solution yields $t(a_{1}^{*}{)}^{2}=0$ and, therefore, cannot be the supremum. The second solution yields

$$\begin{array}{l} t(a_{2}^{*}{)}^{2}=n\frac{A}{B}. \end{array}$$

By the Cauchy Schwarz inequality, (*E*[*X**Z*])^2^≤*E*[*Z*^2^]*E*[*X*^2^], but equality is ruled out by Assumption 1(ii). Since *A*≥0 and *E*[*Y*^2^] > 0, we have *B* > 0. It remains to show that this is indeed the supremum. For *β* = 0, the t-ratio equals zero and is independent of the weight *a*. Therefore, we can restrict attention to the case *β*≠0. First, consider the second derivative at the postulated maximizer:

$$\begin{array}{l} {\frac{\partial^{2}}{\partial a^{2}}t(a{)}^{2}|}_{a=a_{2}^{*}}=-\frac{2E[Y^{2}]C^{4}}{AB^{2}}<0. \end{array}$$

The inequality follows because *A* > 0 whenever *β*≠0, otherwise Var(Z−λX)=0 for λ=E(ZY)/E(XY) which is ruled out by Assumption 1(ii). Also, *B* > 0, *C*≠0, and Assumption 1(i) guarantees that *E*(*Y*^2^) > 0. Therefore, $a_{2}^{*}$ is a local maximizer. Since the t-ratio is a continuously differentiable function, $a_{2}^{*}$ is also a global maximizer of the squared t-ratio as long as the squared t-ratio’s limit as a→±∞ is smaller than (or equal to) its value at $a_{2}^{*}$. One can show that the two limits are equal and that they are smaller than (or equal to) the squared t-ratio at $a_{2}^{*}$ if the following condition holds:

(B4) $$\begin{array}{l} -\frac{E[Y^{2}]C^{2}}{BD}\geq 0 \end{array}$$

where

$$\begin{array}{l} D:=(E[Y(X-Z)]{)}^{2}-E[Y^{2}]E[(X-Z{)}^{2}]. \end{array}$$

By the Cauchy-Schwarz inequality, we have *D*≤0, but equality is ruled out by Assumption 1(iii), so *D* < 0. *D* < 0 and *B* > 0 then imply that (B4) holds and we thus have shown that $a_{2}^{*}$ is indeed the global maximizer of the squared t-ratio when *C*≠0. We now turn to the case *C* = 0. In this case, the first-order condition (B3) has only one solution,

$$\begin{array}{l} a_{3}^{*}:=\frac{E(ZY)\left[E(XZ)E(ZY)-E(XY)E(Z^{2})\right]}{E(XY)(E(XY)-2E(ZY))E(Z^{2})+(2(XZ)-E(X^{2}))(EZY{)}^{2}}, \end{array}$$

which, when substituted back into the t-ratio yields $t(a_{3}^{*})=0$ and therefore cannot be a maximizer. Therefore, the maximum of the t-ratio is

$$\begin{array}{l} \lim_{a\to \infty }t(a{)}^{2}=\lim_{a\to -\infty }t(a{)}^{2}=-\frac{(E[(X-Z)Y]{)}^{2}}{D}>0 \end{array}$$

which is also equal to $t(a_{2}^{*}{)}^{2}$ because $a_{2}^{*}=\infty$ when *C* = 0. In conclusion, $a_{2}^{*}$ is the global maximizer of the squared t-ratio for any value of *C*. The expression for $t(a_{2}^{*})$ follows simply by evaluating the t-ratio at the global maximizer and some straightforward algebra. ▪

Proof of Theorem 2. The t-ratio can be written as

$$\begin{array}{l} \bar{t}(\omega )=\frac{\tilde{\beta }(\omega )}{\bar{\sigma }(\omega )}=\frac{E[\tilde{W}(\omega_{1},\omega_{2})\tilde{Y}]}{\sqrt{E[\tilde{\varepsilon }(\omega {)}^{2}\tilde{W}(\omega_{1},\omega_{2}{)}^{2}]}}. \end{array}$$

Define $\tilde{\omega }=\left(\frac{\omega_{1}}{\omega_{1}+\omega_{2}},\frac{\omega_{2}}{\omega_{1}+\omega_{2}},\frac{\omega_{3}}{\omega_{3}+\omega_{4}},\frac{\omega_{4}}{\omega_{3}+\omega_{4}}\right)$. Then,

$$\begin{array}{ll} \tilde{\varepsilon }(\tilde{\omega }) & =\tilde{Y}-\tilde{\beta }(\tilde{\omega })\tilde{W}\left(\frac{\omega_{3}}{\omega_{3}+\omega_{4}},\frac{\omega_{4}}{\omega_{3}+\omega_{4}}\right)\\ & =\tilde{Y}-\frac{E\left[\frac{\tilde{W}(\omega_{1},\omega_{2})}{\omega_{1}+\omega_{2}}\tilde{Y}\right]}{E\left[\frac{\tilde{W}(\omega_{1},\omega_{2})}{\omega_{1}+\omega_{2}}\frac{\tilde{W}(\omega_{3},\omega_{4})}{\omega_{3}+\omega_{4}}\right]}\frac{\tilde{W}(\omega_{3},\omega_{4})}{\omega_{3}+\omega_{4}}\\ & =\tilde{Y}-\tilde{\beta }(\omega )\tilde{W}(\omega_{3},\omega_{4})\\ & =\tilde{\varepsilon }(\omega ) \end{array}$$

and therefore it is obvious that

$$\begin{array}{l} \bar{t}(\tilde{\omega })=\frac{\frac{E[\tilde{W}(\omega_{1},\omega_{2})\tilde{Y}]}{\omega_{1}+\omega_{2}}}{\sqrt{E\left[\tilde{\varepsilon }(\omega {)}^{2}{\left[\frac{\tilde{W}(\omega_{1},\omega_{2})}{\omega_{1}+\omega_{2}}\right]}^{2}\right]}}=\frac{E[\tilde{W}(\omega_{1},\omega_{2})\tilde{Y}]}{\sqrt{E[\tilde{\varepsilon }(\omega {)}^{2}\tilde{W}(\omega_{1},\omega_{2}{)}^{2}]}}=\bar{t}(\omega ). \end{array}$$

Define that $a_{1}:=\frac{\omega_{1}}{\omega_{1}+\omega_{2}}$ and $a_{2}:=\frac{\omega_{3}}{\omega_{3}+\omega_{4}}$. Then,

$$\begin{array}{l} \bar{t}(\omega )=\bar{t}(a_{1},1-a_{1},a_{2},1-a_{2}). \end{array}$$

▪

The following lemma is a slightly modified version of the results in Black et al. (2000):

Lemma 2.

1. *Under Assumptions 2, 3, and 5, if* *β*≥0,
  (B5) $$\begin{array}{l} \max_{a=0,1}\beta_{OLS}(a)\leq \beta , \end{array}$$
  *and, if* *β*≤0 *, then*
  (B6) $$\begin{array}{l} \beta \leq \min_{a=0,1}\beta_{OLS}(a). \end{array}$$
2. *Under Assumptions 2, 3, and 6, if* *β*≥0,
  (B7) $$\begin{array}{l} \beta \leq \min_{a=0,1}\beta_{OLS-INV}(a), \end{array}$$
  *and, if* *β*≤0 *, then*
  (B8) $$\begin{array}{l} \max_{a=0,1}\beta_{OLS-INV}(a)\leq \beta . \end{array}$$
3. *Under Assumptions 2 and 4, if* *β*≥0,
  (B9) $$\begin{array}{l} \beta \leq \min_{a=0,1}\beta_{IV}(a,1-a), \end{array}$$
  *and, if* *β*≤0 *, then*
  (B10) $$\begin{array}{l} \max_{a=0,1}\beta_{IV}(a,1-a)\leq \beta . \end{array}$$

Proof. First, notice that by Assumption 2,

(B11) $$\begin{array}{l} \beta_{OLS}(a)=\beta \cdot \frac{E[X^{*}W(a)]}{E[X^{*}W(a)]+E[U(a)W(a)]} \end{array}$$

where U(a):=aU+(1−a)V. By Assumptions 3 and 5, $E[X^{*}W(a)]>0$ and *E*[*U*(*a*)*W*(*a*)]≥0 for a=0,1, so

$$\begin{array}{l} \frac{E[X^{*}W(a)]}{E[X^{*}W(a)]+E[U(a)W(a)]}\leq 1 \end{array}$$

and the lower bound in (B7) and the upper bound in (B8) follow. Now, notice that Assumption 2 also implies

(B12) $$\begin{array}{l} \beta_{OLS-INV}(a)=\frac{\beta^{2}E[(X^{*}{)}^{2}]+E[\varepsilon^{2}]}{\beta E[X^{*}W(a)]} \end{array}$$

By Assumptions 3 and 6, if *β*≥0, then

$$\begin{array}{l} \beta_{OLS-INV}(a)\geq \frac{\beta^{2}E[(X^{*}{)}^{2}]}{\beta E[X^{*}W(a)]}\geq \beta , \end{array}$$

yielding the upper bound in (B7). The lower bound in (B8) for *β*≤0 follows from the same argument by reversing the above inequalities. Now, we show that the IV bounds hold. Notice that by Assumption 2,

(B13) $$\begin{array}{l} \beta_{IV}(a,1-a)=\beta \cdot \frac{E[X^{*}W(1-a)]}{E[W(a)W(1-a)]}. \end{array}$$

Assumption 4 then implies that, for a=0,1, the numerator and denominator are both positive and the latter is not larger than the former, so the IV bounds (B9) and (B10) follow. ▪

Proof of Theorem 3. To simplify the presentation, we use the notation *σ*_*A**B*_ for *E*[*A**B*] and $\sigma_{A}^{2}$ for *E*[*A*^2^], where *A* and *B* are random variables. Consider the first statement of the theorem. First, notice that the OLS bound holds for a∈{0,1} by Lemma 2. From the proof of Lemma 2, we know that *β*_*O**L**S*_(*a*)≤*β* holds if, and only if,

(B14) $$\begin{array}{l} \frac{E[X^{*}W(a)]}{E[X^{*}W(a)]+E[U(a)W(a)]}\leq 1. \end{array}$$

To show that this inequality holds only for a∈{0,1} and not for any other value of *a*, we assume that a∉{0,1} and then construct a data-generating process characterized by

$$\begin{array}{l} \left(\sigma_{\varepsilon X^{*}},\sigma_{\varepsilon U},\sigma_{\varepsilon V},\sigma_{X^{*}}^{2},\sigma_{U}^{2},\sigma_{V}^{2},\sigma_{UV},\sigma_{X^{*}U},\sigma_{X^{*}V}\right) \end{array}$$

which satisfies all assumptions of the theorem but violates (B14). Suppose 0<a<1. Let *ϵ* > 0 and $\sigma_{U}^{2}>0$. Set $\sigma_{UV}=\sigma_{X^{*}U}=\sigma_{\varepsilon X^{*}}=\sigma_{\varepsilon U}=\sigma_{\varepsilon V}=0$, $\sigma_{X^{*}}^{2}=\sigma_{V}^{2}=a\sigma_{U}^{2}/(1-a)+\epsilon$, and $\sigma_{X^{*}V}=-\sigma_{V}^{2}+a\epsilon /2$. Then Assumption 2 holds. Since $\sigma_{X^{*}V}+\sigma_{UV}\leq 0$, $\sigma_{X^{*}U}+\sigma_{UV}\leq 0$, and

$$\begin{array}{l} E[XZ]=\sigma_{X^{*}}^{2}+\sigma_{X^{*}U}+\sigma_{X^{*}V}+\sigma_{UV}=\frac{a\epsilon }{2}>0, \end{array}$$

Assumption 4 holds, which implies Assumption 3. Assumption 5 is satisfied by definition of the above quantities. We also have

$$\begin{array}{l} E[X^{*}W(a)]=aE[X^{*}X]+(1-a)E[X^{*}Z]>0. \end{array}$$

By the definition of $\sigma_{V}^{2}$,

$$\begin{array}{l} \sigma_{V}^{2}=\frac{a^{2}\sigma_{U}^{2}+(1-a{)}^{2}\sigma_{V}^{2}}{1-a}+a\epsilon , \end{array}$$

so that

$$\begin{array}{l} \left(1-a)\sigma_{X^{*}V}<-(a^{2}\sigma_{U}^{2}+(1-a{)}^{2}\sigma_{V}^{2}\right) \end{array}$$

and thus

$$\begin{array}{l} E[U(a)W(a)]=a\sigma_{U}^{2}+(1-a{)}^{2}\sigma_{V}^{2}+(1-a)\sigma_{X^{*}V}<0. \end{array}$$

Therefore, (B14) is violated. Suppose *a* < 0. Let *ϵ* > 0, *δ* > 0, and $\sigma_{U}^{2}>0$. If a<−2, then pick *δ* small enough so that δ≤−4ϵ/(2+a). Set $\sigma_{X^{*}U}=\sigma_{\varepsilon X^{*}}=\sigma_{\varepsilon U}=\sigma_{\varepsilon V}=0$, $\sigma_{UV}=-a\sigma_{U}^{2}/(2(1-a))+\epsilon$, $\sigma_{V}^{2}=2\sigma_{UV}+a\sigma_{U}^{2}/(1-a)-\delta$, $\sigma_{X^{*}V}=-\sigma_{V}^{2}-\delta a/2$, and $\sigma_{X^{*}}^{2}=\sigma_{V}^{2}(1-a)$. Then Assumption 2 holds. Assumption 3 holds because $E[XX^{*}]=\sigma_{X^{*}}^{2}+\sigma_{X^{*}U}=\sigma_{X^{*}}^{2}>0$ and

$$\begin{array}{l} E[ZX^{*}]=\sigma_{X^{*}}^{2}+\sigma_{X^{*}V}=\sigma_{V}^{2}(1-a)-\sigma_{V}^{2}-\frac{\delta a}{2}>0. \end{array}$$

Assumption 5 is satisfied by definition of the above quantities. We also have

$$\begin{array}{l} E[X^{*}W(a)]=\sigma_{X^{*}}^{2}+a\sigma_{X^{*}U}+(1-a)\sigma_{X^{*}V}=\sigma_{X^{*}}^{2}-(1-a)\sigma_{V}^{2}-\frac{(1-a)a\delta }{2}=-\frac{(1-a)a\delta }{2}>0. \end{array}$$

By the definition of $\sigma_{V}^{2}$,

$$\begin{array}{l} \sigma_{V}^{2}=\frac{a^{2}\sigma_{U}^{2}+(1-a{)}^{2}\sigma_{V}^{2}+2a(1-a)\sigma_{UV}}{1-a}-a\delta , \end{array}$$

so that

$$\begin{array}{l} \left(1-a)\sigma_{X^{*}V}<-(a^{2}\sigma_{U}^{2}+(1-a{)}^{2}\sigma_{V}^{2}+2a(1-a)\sigma_{UV}\right) \end{array}$$

and thus

$$\begin{array}{l} E[U(a)W(a)]=a\sigma_{U}^{2}+(1-a{)}^{2}\sigma_{V}^{2}+2a(1-a)\sigma_{UV}+(1-a)\sigma_{X^{*}V}<0. \end{array}$$

Therefore, (B14) is violated. In the case *a* > 1, we can construct a data-generating process violating (B14) in a similar fashion as in the case *a* < 0. Now, consider the second statement of the theorem. From the proof of Lemma 2, we know that the inverse OLS bound holds if, and only if,

(B15) $$\begin{array}{l} \frac{\beta^{2}E[(X^{*}{)}^{2}]+E[\varepsilon^{2}]}{\beta E[X^{*}W(a)]}\geq \beta . \end{array}$$

This inequality holds for all a∈[0,1] by the same reasoning as in the proof of Lemma 2 because $E[X^{*}W(a)]>E[(X^{*}{)}^{2}]$ for all a∈[0,1]. To show that (B15) does not hold for any *a* outside the interval [0, 1], we construct a data-generating process that satisfies all assumptions of the theorem but violates (B15). If *a* < 0, pick $\sigma_{X^{*}}^{2}>0$, $\sigma_{X^{*}U}\leq 0$, $\sigma_{X^{*}V}\leq 0$, such that $E[X^{*}X]>(1-1/a)E[X^{*}Z]$, $\sigma_{X^{*}}^{2}+\sigma_{X^{*}U}>0$, and $\sigma_{X^{*}}^{2}+\sigma_{X^{*}V}>0$. If *a* > 1, then pick $\sigma_{X^{*}}^{2}>0$, $\sigma_{X^{*}U}\leq 0$, $\sigma_{X^{*}V}\leq 0$ such that $E[X^{*}X]<(1-1/a)E[X^{*}Z]$, $\sigma_{X^{*}}^{2}+\sigma_{X^{*}U}>0$, and $\sigma_{X^{*}}^{2}+\sigma_{X^{*}V}>0$. In both cases, Assumptions 3 and 6 hold and Assumption 2 can, as above, be satisfied by simply choosing the three covariances to be zero. However,

$$\begin{array}{l} E[X^{*}W(a)]=aE[XX^{*}]+(1-a)E[ZX^{*}]<0 \end{array}$$

and thus (B15) is violated. Finally, consider the third statement of the theorem. The IV bound holds if, and only if,

(B16) $$\begin{array}{l} \frac{E[X^{*}W(1-a)]}{E[W(a)W(1-a)]}\geq 1. \end{array}$$

Suppose 0<a<1. Set $\sigma_{UV}=\sigma_{X^{*}V}=\sigma_{X^{*}U}=\sigma_{\varepsilon X^{*}}=\sigma_{\varepsilon U}=\sigma_{\varepsilon V}=0$ and let $\sigma_{U}^{2},\sigma_{V}^{2},\sigma_{X^{*}}^{2}$ any positive values. Then Assumptions 2 and 4 hold. However,

$$\begin{array}{l} E[X^{*}W(1-a)]=\sigma_{X^{*}}^{2}+E[X^{*}U(1-a)]=\sigma_{X^{*}}^{2}>0 \end{array}$$

and

$$\begin{array}{l} E[U(a)W(1-a)]=a(1-a)(\sigma_{U}^{2}+\sigma_{V}^{2})>0, \end{array}$$

which means the bound (B16) is violated. Suppose *a* < 0. Set $\sigma_{UV}=\sigma_{X^{*}V}=\sigma_{\varepsilon X^{*}}=\sigma_{\varepsilon U}=\sigma_{\varepsilon V}=0$. Pick $\sigma_{U}^{2},\sigma_{V}^{2}>0$ and $\sigma_{X^{*}U}$ small enough so it satisfies $\sigma_{X^{*}U}<-(1-a)(\sigma_{U}^{2}+\sigma_{V}^{2})$. Choose $\sigma_{X^{*}}^{2}>-(1-a)\sigma_{X^{*}U}$. Then Assumptions 2 and 4 are satisfied because

$$\begin{array}{l} E[XZ]>a\sigma_{X^{*}U}>0 \end{array}$$

and $\sigma_{X^{*}U}+\sigma_{UV}\leq 0$ and $\sigma_{X^{*}V}+\sigma_{UV}\leq 0$. However,

$$\begin{array}{l} E[X^{*}W(1-a)]=\sigma_{X^{*}}^{2}+(1-a)\sigma_{X^{*}U}+a\sigma_{X^{*}V}>0 \end{array}$$

and

$$\begin{array}{l} E[U(a)W(1-a)]=a(1-a)(\sigma_{U}^{2}+\sigma_{V}^{2})+a\sigma_{X^{*}U}>0, \end{array}$$

which means, again, the bound (B16) is violated. In the case *a* > 1, such a violation of (B16) can be created in a similar fashion. ▪

## Appendix C simulations without nondifferentiability

Nondifferentiability of measurement errors and exogeneity of *X*^*^ is required for $\beta_{OLS}=\beta_{IV}=0$ to be a testable implication of the null of no effect. If either of these conditions is violated, the null stated in (4) is no longer equivalent to β=0. To investigate the robustness properties of our test, we conduct further simulations in which we allow one measurement error, *V*, to be positively or negatively correlated with *ε*. Other than nondifferentiability, the simulation design employed here closely mirrors that of Section 4. Table B1 displays the parameter values used in each scenario.

**Table B1.** Parameter values in each scenario.

Scenario	*σ*_*V* *ε*_	$\sigma_{U}^{2}$, $\sigma_{V}^{2}$	*σ*_*U* *V*_	$\sigma_{X^{*}U}$	$\sigma_{X^{*}V}$
1	0.05	2	0	−0.3 or −0.7	−0.3 or −0.7
2	0.05	2	0.5	−0.3 or −0.7	−0.3 or −0.7
3	0.05	2	−0.5	−0.3 or −0.7	−0.3 or −0.7
4	−0.05	2	0	−0.3 or −0.5	−0.3 or −0.5
5	−0.05	2	0.5	−0.3 or −0.5	−0.3 or −0.5
6	−0.05	2	−0.5	−0.3 or −0.5	−0.3 or −0.5

Our test is shown to be robust to *small* deviation from nondifferentiability. As illustrated in Figs. B1, the simulation results exhibit analogs patterns to those presented in Section 4. A slight size distortion is observed across the scenarios. The rejection frequencies under the null (*β* = 0) are reported in Table B2. Only the standard t-test based on the OLS of *Y* on *X* (a contaminated measurement with nondifferential ME) controls the size around the nominal level. This is naturally the case because $\underset{\omega \in R}{\sup}|\beta (\omega )|$ deviates from 0 without nondifferentiability.

**Table B2.** Null rejection probabilities in the six different scenarios.

$\sigma_{X^{*}U}$	$\sigma_{X^{*}V}$	Test	S1	S2	S3	S4	S5	S6
strong	strong	Oracle	0.070	0.053	0.101	0.082	0.066	0.098
		OLS w/ x	0.038	0.045	0.048	0.050	0.054	0.052
		OLS w/ z	0.085	0.092	0.074	0.091	0.079	0.095
		IV x|z	0.033	0.005	0.065	0.036	0.002	0.085
		tmax	0.076	0.074	0.097	0.087	0.081	0.105
strong	weak	Oracle	0.072	0.075	0.089	0.064	0.059	0.071
		OLS w/ x	0.048	0.055	0.046	0.043	0.045	0.041
		OLS w/ z	0.077	0.073	0.076	0.072	0.064	0.085
		IV x|z	0.001	0.037	0.038	0.003	0.033	0.045
		tmax	0.077	0.076	0.082	0.072	0.061	0.077
weak	strong	Oracle	0.067	0.064	0.075	0.068	0.066	0.073
		OLS w/ x	0.048	0.042	0.059	0.066	0.059	0.044
		OLS w/ z	0.080	0.091	0.070	0.095	0.100	0.085
		IV x|z	0.001	0.046	0.038	0.000	0.040	0.038
		tmax	0.085	0.084	0.084	0.088	0.088	0.072
weak	weak	Oracle	0.066	0.068	0.052	0.060	0.057	0.060
		OLS w/ x	0.050	0.060	0.043	0.032	0.082	0.057
		OLS w/ z	0.089	0.085	0.072	0.074	0.058	0.074
		IV x|z	0.017	0.061	0.002	0.011	0.072	0.001
		tmax	0.084	0.088	0.067	0.066	0.072	0.074

The values of $\sigma_{X^{*}U}$ and $\sigma_{X^{*}V}$ vary between “strong” (−0.7 for scenarios 1–3; −0.5 for scenarios 4–6) and “weak” (−0. 3 for all scenarios)

This robustness property may, of course, be lost for other data-generating processes in which both MEs are differential or *X*^*^ is endogenous. In general, the strength of the correlation between MEs and *ε* or between *X*^*^ and *ε* determines the extent of the distortion in size and power. This additional exercise clearly shows that the nondifferentiability of measurement errors is crucial to guarantee the validity of our test.
